# Decolonising global health evaluations: A scoping review of methodological frameworks

**DOI:** 10.1371/journal.pgph.0007362

**Published:** 2026-09-25

**Authors:** Sali Hafez, Agata Pacho, Seyi Soremekun, Meghna Ranganathan, Mitzy Gafos, Lucy Platt, Ruth Ponsford

**Affiliations:** 1 Centre for Evaluation, Faculty of Public Health and Policy, London School of Hygiene & Tropical Medicine (LSHTM), London, United Kingdom; 2 Department of Sociology, Purdue University, West Lafayette, Indiana, United States of America; 3 Faculty of Public Health and Policy, London School of Hygiene & Tropical Medicine (LSHTM), London, United Kingdom; 4 Centre for Evaluation, Faculty of Epidemiology and Population Health, London School of Hygiene & Tropical Medicine (LSHTM), London, United Kingdom; PLOS: Public Library of Science, UNITED STATES OF AMERICA

## Abstract

The field of global health evaluation is critiqued for its reliance on Eurocentric paradigms that perpetuate epistemic injustice. A rapidly evolving yet conceptually fragmented literature on decolonising evaluation has emerged in response. We conducted a scoping review to chart the nature and breadth of this literature and synthesise insights to guide future evaluation scholarship and practice. Academic database searches identified 1,024 records, of which 732 were screened at title and abstract following deduplication. Combined with 138 records from grey literature and citation searches, a total of 653 reports were assessed at full text, from which 59 records were included in the final synthesis. Our synthesis reveals that the literature points in two concurrent, dialectical and complementary theoretical projects: the first involving the deconstruction of colonial systems, and the second the (re)construction of decolonial and Indigenous-centred alternatives. We organised our findings into a typology that illustrates a spectrum of praxis in global public health evaluation, which moves from minimal adaptation of dominant methodological approaches to a more profound philosophical and political reorientation of practice. This paper calls for greater conceptual precision in the description of chosen approaches to prevent the co-optation of decolonial language and the need for empirical research to bridge the gap between theory and practice.

## Introduction

*‘So as Ezeulu urinated outside the guardroom his eyes looked for the new moon. But the sky had an unfamiliar face. It was impossible to put one’s finger anywhere on it and say that the moon would come out there. A momentary alarm struck Ezeulu but on thinking again he saw no cause for alarm. Why should the sky of Okperi be familiar to him? Every land had its own sky; it was as it should be.’*

Chinua Achebe, Arrow of God.

The above quote comes from a story by Chinua Achebe [1] about Ezeulu, the Igbo chief priest, who, over the course of the novel finds himself alienated while away from home in Okperi. He panics momentarily when he goes to observe the new moon, as the sky looks unfamiliar. Ezeulu then realises that the sky in Okperi must look different from the one in his home, Umuaro, he accepts both that difference and the limits of his own understanding.

This moment of recognition that different places may require different ways of seeing that may be inaccessible to the foreign eye offers a powerful metaphor for global health. Such thoughtfulness may be lacking among researchers and practitioners who continue to uncritically apply theory and methods derived from a ‘Eurocentric’ position, regardless of which ‘sky’ they are looking at. This lack of consideration is not inconsequential. Instead, during Europe’s colonial period, global health, through the use of ‘Eurocentric’ frameworks, was able to *‘aid and excuse the dispossession that created, widened or entrenched inequity’* [2] (Page 18). In other words, global health has served as a means of what has been described as a form of epistemic injustice perpetrated against the knowledge and knowledge systems of Indigenous people, the colonised, racialised and historically marginalised. As Abimbola (2024) [2]contends, global health research continues to cater to external audiences, particularly from Europe and North America, rather than prioritising the needs and perspectives of local communities. Consequently, it produces knowledge that *‘at best has the relevance of a cliché, typically appealing to the foreign gaze’* (Page 41).

At the same time, calls for epistemic justice in global health, understood as equitable recognition of the value of different forms of knowledge and ways of knowing, accompanied by a tangible shift in epistemic power to local actors, have been recognised as essential to the production of knowledge which is of real utility [2–4]. In recent years the global health community has been forced to interrogate its historical colonial roots and how related power dynamics continue to influence decision-making [3], leadership [5], distribution of resources [6,7], knowledge production [8], educational curricula [9,10], publishing [11], and design and evaluation of interventions in low- and middle-income countries [12]. Debates about what level and type of changes to practice, education and wider scholarship are necessary and appropriate are ongoing. Some scholars prioritise scholarships on epistemological and ontological shifts [13], while others advocate for other institutional approaches [14] framing ‘equity, diversity and inclusion (EDI)’, ‘equitable partnerships’ or ‘localisation’ as constitutive of a decolonial agenda. Alongside these debates, concerns about tokenism, self-serving agendas of Global Northern institutions, and performative engagement are increasingly voiced [15,16]. Recent calls to ‘decolonise the decolonisation movement’ highlight ongoing power imbalances within global health and the Global North’s continued influence and co-optation of these debates that are manifesting [17]. We share these concerns and argue elsewhere, drawing on implementation science as an example of a global health area that must move beyond a focus on equity toward a more disruptive decolonial approach- one that critically examines its methodological and epistemological foundations [18].

To anchor the discussion that follows, we adopt working definitions drawn from the from the decolonial and Indigenous literature, while acknowledging their diversity and contestation. Decolonisation is most familiar as the formal political liberation and independence of colonised nations from formal colonial rule. However, decolonial scholarship holdsthat independence did not dissolve colonial relations of power, which persist as coloniality: the enduring colonial ordering of knowledge, economy and institutions that outlives the formal end of empire [19]. As such we use ‘decolonising’ in its processual sense, as the ongoing dismantling of these colonial relations within knowledge production, including the power to define what counts as valid knowledge, to set the terms of value, and to control knowledge production [20]. Decolonising evaluation follows from this, as a practice that interrogates and redistributes epistemic power and challenges epistemic injustice. Decolonising evaluation can thus be read as a sustained response to that injustice within the field. Indigenous-centred approaches, in turn, denote evaluation grounded in, and accountable to, the worldviews, protocols and sovereignty of specific Indigenous communities [20,21]. We treat these terms as contested and evolving and return to that contestation in different sections of this paper.

Evaluation has been described as having the potential and power to become one of the *‘worst instruments of epistemological imperialism’* [22]. Historically, the need for evaluation, its methods and outputs have been shaped by colonial and imperial interests. Today, overreliance on ‘Eurocentric’ frameworks, insufficient scrutiny of the power dynamics within the field and its associations with international development continue to perpetuate epistemic injustices. In response, a large, diverse and rapidly expanding body of literature has emerged to address these concerns and advance decolonial and Indigenous approaches to evaluation. However, within global health, the core principles of this literature and implications for broader evaluation practice remain poorly synthesised and understood by scholars, evaluators and practitioners. Recognising this gap, and as part of the Centre for Evaluation at the London School for Hygiene and Tropical Medicine (LSHTM)’s commitment to strengthening evidence-informed evaluation practice, we conducted a scoping review of evaluation methods rooted in decolonial and Indigenous approaches. Building on Pant et al.(2022) [23]’s synthesis of decolonising global health evaluation and Stefanski et al.(2025) [24]’s scoping of Indigenous evaluation methods and guiding principles, we extend current syntheses by mapping a rapidly evolving yet conceptually fragmented field of literature and developing an analytical typology that organises the literature along a spectrum of epistemic transformation, from minimal cultural adaptation to profound philosophical and political reorientation. Thereby it fosters an engagement with their theoretical and methodological foundations and to consider the implications of this body of work for evaluation research and practice. At the same time, we attend to the tensions that arise between these foundations and the structural constraints that shape global health evaluation practice. Moreover, echoing the reflexive stance of Chambers et al. [25], as part of our process, we critically examine the dissonances between the topic of the study (the literature), and the methodology of the scoping review, recognising the ways in which the method constrains engagement with decolonial and Indigenous principles in our own practice.

## Methods

### Ethics statement

Ethical approval was not required for this study as it is a scoping review of existing literature and did not involve the collection of primary data or engagement with human participants. All sources were publicly available and appropriately cited in accordance with academic and research integrity standards.

### Objective and questions

The primary objective of this scoping review is to analyse methodological literature and frameworks for decolonial evaluation in global health. As there is limited systematic exploration of this literature, our aim was to chart the breadth and nature of the available work and practical guidance, identifying its conceptual and theoretical underpinnings, as well as implications for methods and practice. We also sought to identify the tensions and challenges to implementing such approaches identified by this body of literature. Accordingly, our three main research questions were:

1. What are the core concepts and theoretical orientations that underpin decolonial and Indigenous frameworks for evaluation in global health?
2. What specific methodological and practical approaches for conducting decolonial and Indigenous evaluations are described?
3. What are the identified challenges or tensions related to the application of decolonial and Indigenous evaluation methodologies?

### Theoretical framework and approach

Our broad perspective was informed by two interconnected sets of theory. First, decolonial theory, which moves beyond critique of *historical* power imbalances to actively interrogate the *current* ‘Eurocentrism’, epistemic injustice, and the privileging of ‘Eurocentric’ knowledge systems, as discussed by Chilisa and Mertens [26]. From this perspective, decoloniality is understood as a transformative epistemological project tied to the intellectual sovereignty of Indigenous people and colonised communities. Second, Indigenous evaluation and research paradigms which we conceptualise as inherently de/anticolonial in approach, but also as existing in their own right not only in opposition to ‘Eurocentric’ methodologies. Both paradigms emphasise relational accountability and the Four Rs (respect, relevance, reciprocity, and responsibility) [27,28], guiding us in this review to honour knowledge holders, ensure relevance of research and method, reciprocate through useful synthesis, and taking responsibility for interpretation.

Decolonial and Indigenous principles shaped all stages of this review, as detailed in the following sections. In the study design, these guided us to frame questions and approaches around decolonial philosophies, power, and ethics, while deliberately including grey literature to resist favouring peer-reviewed sources and to incorporate diverse work by Indigenous scholars and community knowledge. The study followed Arksey and O’Malley framework for scoping reviews [29], the seven-step guidance by Peters et al. (2015) [30], and the Preferred Reporting Items for Systematic Reviews and Meta-analysis extension for Scoping Reviews (PRISMA-ScR) [31] (S3 Table). This approach was utilised to promote rigour and transparency, while allowing the flexibility to refine the search process iteratively and incorporate diverse literature on ways of knowing and doing. We actively interrogated the positivistic underpinnings common in conventional scoping reviews which, as Chambers et al. (2018) [25] argue, often prioritise ‘procedural objectivity’ where methodological rigour is sought through strict adherence to protocols that aim to reduce bias and enhance replicability. In practice, this plays out as a reductionist process that often suppresses the interpretive depth, tacit knowledge, and dialogic interactions. Instead of claiming the positivist ideal of a ‘neutral’ researcher, we utilised critical reflexivity to treat the review as an embodied ‘dialogue’ between the research team ensuring that our synthesis remained grounded in relationality and specific attention to interdependence (who is connected to whom, under what conditions, and with what consequences). Part of this dialogue involves an engagement with terms used in the literature (e.g., decolonial, anti-colonial, Indigenous, aboriginal, equitable) acknowledging that such concepts are often problematic and can be indicative of colonial discourse. In this paper, we use the terms ‘Western’, ‘Global South’, and ‘Global North’ to highlight the geopolitical dimensions of historical economic and political power disparities. The term ‘Eurocentric’ is used to underscore the philosophical roots of dominant methodological traditions in global health evaluation.

We convened a study Advisory Group comprising four experts from LSHTM in the fields of global health, decolonial theory and decolonial feminist evaluation to provide experienced input from their respective areas of expertise. Guidance from the Advisory Group informed our navigation of the literature and theory by reviewing the study protocol, helping to refine conceptual definitions, and providing critical feedback on emerging findings. While we acknowledge and thank the decolonial and Indigenous scholars whose knowledge informs this work, the absence of Indigenous scholars among the co-authors or Advisory Group remains a limitation reflecting the structures we critique.

### Search strategy

Recognising that decolonial and Indigenous scholarship often uses diverse terminologies and relevant literature may be underrepresented in some bibliographic databases, we used a multi-staged process for our literature search.

**Fig 1:**
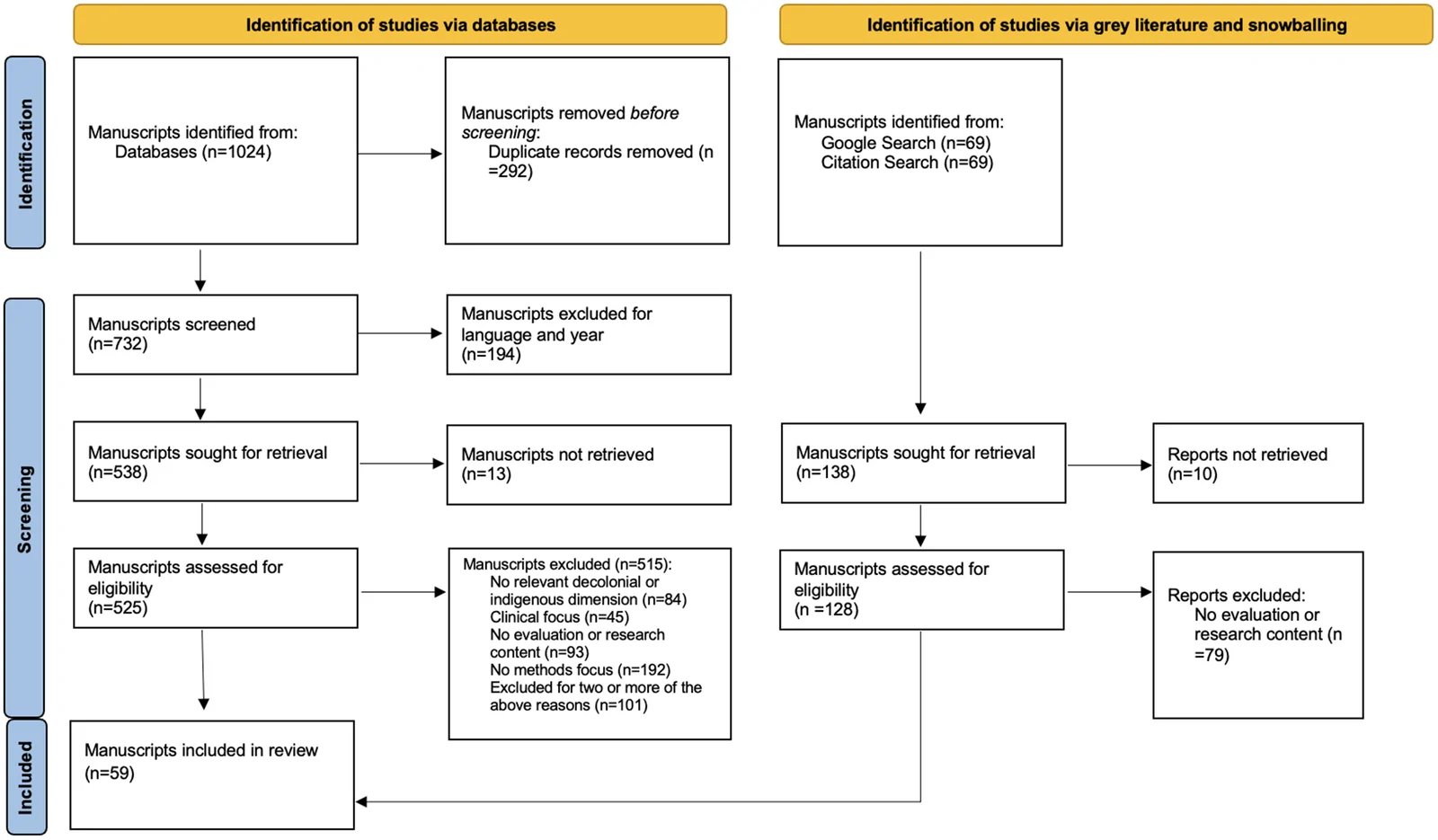
PRISMA 2020 flow chart for reporting scoping review.

An initial search strategy was developed using combination of search terms across four domains: 1) evaluation, 2) global health 3) decolonisation and Indigenous knowledge, and 4) methodological frameworks (S1 Table), including Boolean operators to conduct an extensive search of peer-reviewed databases (Medline Ovid, Global health and Embase chosen to reflect published literature in the global health field) and grey literature. The initial search terms were identified through a rapid review of the literature. Following an initial search of the literature that yielded few results, terms were then broadened beyond global health to include international development following discussion between the review team (S1 Table). This search process resulted in a higher number of results, but when screened and reviewed, resulted in few relevant manuscripts (Fig 1), a finding we interpret as an indication of limited representation of decolonial and Indigenous evaluation approaches within the global health, and international development literature, while acknowledging that our restriction to English-language publications likely contributed to this paucity, as discussed in the next section. Following consultation with the library team at LSHTM, we also learnt that the discipline-specific terminologies and indexing mechanisms, such as MeSH, often do not recognise or categorise decolonial and Indigenous methodological literature, thereby making them invisible to these usual search strategies [20].

Second, we explored the grey literature by conducting searches on Google, Google Scholar, and targeted websites of evaluation societies, Indigenous research centres, and non-governmental organisations (S2 Table). This was a deliberate methodological choice to honour the nature of Indigenous knowledge, the body of practice-based wisdom, community-generated reports, and theoretical work that exists outside of the peer-review systems, as recommended by Goldman [32].

The database and grey literature searches were limited to manuscripts published in English between January 2005 and February 2026. This time frame was chosen to capture the recent growth in literature in this field as well as key foundational Indigenous and decolonial evaluation frameworks known to the research team and Advisory Group including, for example Kawakami et al. (2007) [33] and LaFrance & Nichols (2008) [34]. Initial database and grey literature searchers were run on 21 June 2024 and were rerun on 12 February 2026 using the same databases, search strings, and grey literature sources as the original review to capture any new literature that emerged during the analysis process (S1 Table and S2 Table).

Lastly, we conducted snowballing through an intentional citation search of included records, expanding the analysis beyond global health and international development evaluation literature to include additional relevant key foundational works where these met our inclusion criteria. Moreover, this intentional search looked for manuscripts from the least represented literature and contexts. This iterative process of ‘following the knowledge’ proved to be the most fruitful method for identifying a coherent body of literature, allowing us to trace intellectual lineages and uncover critical Indigenous scholarship that the initial database search had obscured, as suggested by Goldman et al. (2024) [32].

### Inclusion and exclusion criteria

The inclusion criteria were developed to capture literature that explicitly engaged with the conceptual, theoretical, or methodological dimensions of decolonising evaluation. We sought to include reports that focused on the methodological literature that elucidated paradigms and frameworks themselves, rather than the empirical application of these in a specific case study. The criteria were refined iteratively during the initial screening phase to ensure clarity and consistency among the review team (Table 1).

**Table 1:** Inclusion and exclusion criteria.

	Inclusion	Exclusion
Language	English	Other languages
Publication date	January 2005–12 February 2026	Manuscripts published before or after the included time window
Scope	Must explicitly address methodological aspects of decolonising evaluation research, including conceptual, theoretical, or practical aspects. This includes: 1. Evaluation or research concepts, theories, and conceptual frameworks 2. Evaluation methods, approaches, guidance and toolkits	Empirical research without significant conceptual or methodological discussion.
Type	Peer-reviewed records (peer-reviewed articles, book chapters, dissertations, and theses) of different study designs including reviews. Grey literature (evaluation briefs, guidelines, toolkits, blogposts, repositories, and websites)	Editorials, commentaries, conference abstracts, videos, podcasts, or film.
Geographic focus	Global, any country.	NA.

### Screening

Consistent with our own decolonial approach, the screening process was not assumed to be neutral, with inclusion/exclusion criteria applied as an objective technical filter, but rather as an intentional, responsible action. We understood that the decision to include or exclude a manuscript is a form of epistemic governance; therefore, each stage was conducted with a high degree of reflexivity across two screening stages. The first stage included SH and AP independently screening the title and abstract of all returned manuscripts for relevance, followed by a rapid review of the screening database by SS, MR and RP for another layer of rigour. Recognising that the language used to articulate decolonial and Indigenous concepts was diverse and evolving, SH and AP met regularly to discuss and refine inclusion criteria to ensure consistency in the selection of reports. Discrepancies were resolved involving the wider team.

The second stage involved a full-text review of the manuscripts included based on the initial screening. Discrepancies were again discussed between SH and AP and resolved by involving the wider team where necessary. The focus here was to move beyond the language of the abstract to assess the substance and depth of the manuscript’s engagement with methodological frameworks to decolonise evaluation. We were aware of the risk of inclusion of reports that might use decolonial terminology in a superficial or tokenistic manner [35]. Therefore, as per our inclusion criteria, we critically assessed whether manuscripts engaged sufficiently with Indigenous theories, colonialism and its legacies or offered relevant conceptual or theoretical framework or methodological guidance for decolonising evaluation practice. To be included, a manuscript was required to have a tangible methodological component, such as a specific theoretical framework, a relevant theoretical discussion, or actionable methodological guidance.

### Analysis

We developed a data synthesis tool to map, synthesise and capture the nuances of decolonial scholarship in global health evaluation. Beyond standard bibliographic details, the tool captured data on evaluation focus, methods and frameworks used, theoretical underpinning, and contested questions. As illustrated in the characteristics of included studies (S4_Table), we explicitly mapped the context of each framework, distinguishing between settler-colonial contexts (e.g., Canada, Australia) and post-colonial Global South contexts (e.g., Africa, Asia). This allowed us to trace how specific theoretical lineages, such as Kaupapa Māori or Ubuntu, informed distinct methodological choices. We then conducted a thematic synthesis, to draw findings from across studies together to build a typology of approaches to decolonising evaluation. We used an inductive approach drawing on concepts from the literature to inform the development of our themes. To reduce the risk of reproducing colonial hierarchies when synthesising findings, we assigned greater epistemic focus to Indigenous authors and practitioners writing from within their communities (e.g., ‘First Voice’ accounts) over external evaluators and theorists observing those communities**.** Through iterative reading and discussion and ‘reflexive check-ins’ with the wider team and the Advisory Group, we mapped and synthesised the literature as presented in the next section. To promoteconsistency, the charting tool was piloted on a sample of records by SH and AP and refined before charting the remaining records. SH and AP charted independently and met regularly to compare coding and reconcile domain assignment, with discrepancies and boundary cases resolved through discussion with the wider team and the Advisory Group. Domains were derived inductively from recurrent conceptual and methodological features rather than imposed a priori.

### Researchers’ positionalities

We intentionally foreground our positionality in this paper, understanding that it shapes the assumptions and decisions made at every stage of the review process, and the limitations that flow from them. We write this review as researchers and academics situated within a Global North institution, whose own histories and career trajectories are interwoven with those of the British Empire [36]. Our relationships to that history are not the same. The lead author is Egyptian and reads this scholarship in part through the experience of a country subjected to British colonial occupation. Training in critical post-colonial theory also influences our perspectives and the approach we take. Yet our own education system, regardless of geography, were derived from and accredited by knowledge systems refined during, and for the purposes of, imperial expansion. This presented a paradox during our analysis: naming patterns of epistemic injustice and resistance using the very colonial language (English) and methodological practices (e.g., charting, synthesising) that have been used as a tool to master knowledge and exert epistemic power, ownership, and exclusion. Given our academic and methodological training we are, therefore, unavoidably implicated in the very systems of knowledge production, funding, and publishing that literature included in this review seeks to critique.

Power operates not only between institutions but also among us as co-authors. Our team included members of different levels of seniority, racial and ethnic identities, educational backgrounds, and lived and professional experience across the Global South and North. Our longstanding engagement with global health equity, including the leadership of two co-authors (RP, SH and AP) in LSHTM’s ‘Decolonising Global Health’ and Fighting Against Institutional Racism (FAIR) initiatives, linked this academic exercise with the institutional activities aimed at making changes in teaching, research and evaluations. Our process was strengthened by the LSHTM-based Advisory Group whose expertise in decolonial research, global health equity and anti-racism refined our protocol and interpretations. These experiences and ways of interpretations allowed us to de-centre a singular ‘Eurocentric’ viewpoint from within our own analytical process.

### Accountability statement

This review and its aims reflect our attempts to navigate the contradiction of attempting to decolonise evaluation from within, what hooks (2014) refers to as the ‘belly of the beast’ [37]. Our lived experiences and interest in decolonial research and equity do not resolve this contradiction as the diverse experiences of coloniality are not interchangeable, and proximity to one does not authorise us to systematise the knowledge of peoples living under another, including the ongoing settler colonialism that informs much Indigenous evaluation. We do not claim authority over Indigenous evaluation, especially given the absence of Indigenous co-authorship or advisory governance. Instead, we position the article as a reflexive synthesis directed at the organisations and institutions, we are part of, namely the funders, commissioners, universities and evaluators of the Global North whose practices this scholarship calls to account. Our intended readers are those with the power to change those practices, and the work is accountable first to that task.

In practical terms, we have sought to honour relational accountability [22,26] by foregrounding the words and framings of Indigenous and decolonial scholars rather than translating them into our own, resulting in the use of less uncommon terms in traditional public health literature. This scoping review contributed to developing a reflexive planning guide for the Centre for Evaluation at LSHTM [38], grounding our work practically in the principle of ‘Reciprocity’, a core tenet of the ‘Four Rs’ of Indigenous methodology [28]. Our aim was for this review to generate insights that could inform decolonial efforts within our own institution and be of practical relevance to communities engaged in evaluation. We recognise that accountability of this kind is enacted over time and through relationship and cannot be discharged within one paper.

## Results

The initial search resulted in 1024 records from academic databases. Following the removal of 292 duplicates, 732 records were screened on title and abstract, of which 194 were excluded on the basis of language, publication year, or record type. A total of 538 records were sought for retrieval, of which 13 were not retrievable, leaving 525 reports assessed for eligibility against the inclusion criteria. Of these, 515 were excluded at full-text review for the following reasons: no relevant decolonial or Indigenous dimension (n = 84), clinical focus (n = 45), no evaluation or research content (n = 93), no methods focus (n = 192) or excluded for two or more of the above reasons (n = 101), yielding 10 records from the database search. Simultaneously, grey literature and citation searches identified further 138 records. Of these, 10 were not retrievable and 128 were assessed at full text, of which 79 were excluded for containing no evaluation or research content, yielding 49 records from this arm. Together, the database and grey literature searches produced a total of 59 records included in the final synthesis. The flow of records and specific reasons for exclusion are illustrated in the PRISMA flow diagram (Fig 1).

**Fig 2:**
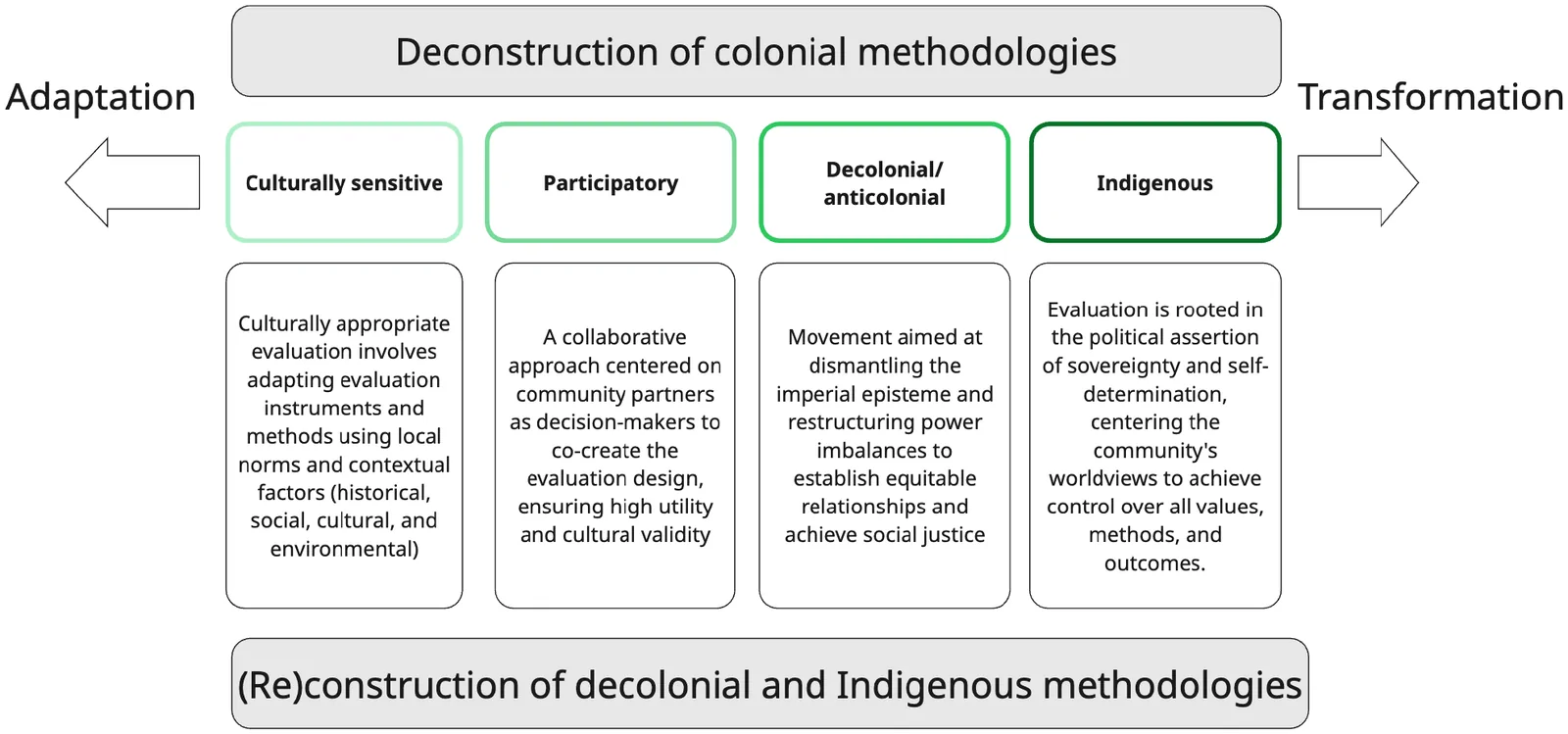
Decolonial evaluation methodologies identified in the literature.

Findings are organised as follows: Part I (Characteristics) describes the scope, composition, temporal trends, and geographical distribution of the included literature. Part II (Deconstruction versus (re)construction) introduces the analytical heuristic (Fig 2) that integrates two distinct but intersecting analytical dimensions. First, the figure captures the nature of the literature as engaging in two concurrent, dialectical and complementary theoretical projects: 1) deconstruction (challenging colonial legacies) and 2) (re)construction of decolonial and Indigenous methodologies. Part III (Typology of decolonial approaches to evaluation) operationalises this heuristic by organising the literature along a spectrum of epistemic transformation, ranging from Indigenous evaluation methodologies and decolonial/anticolonial approaches to participatory and culturally sensitive adaptations, and examines the conceptual foundations and methodological features of each domain. Part IV (Contested questions) synthesises cross-cutting debates that traverse the typology, focusing on evaluator positionality and accountability, tensions between epistemological pluralism and sovereignty, and the practical challenges of implementing decolonial and Indigenous evaluation within global health.

### Part I: Characteristics

The final 59 records included: peer-reviewed articles (n = 42), book chapters (n = 8) and grey literature (n = 9), such as toolkits, guidebooks, and reports from Indigenous organisations and government commissions. The included studies were published between 2007 and 2025, with 53% of records (n = 31) published between 2022 and 2025, suggesting growing momentum and urgency around decolonisation in this field.

The literature is geographically and contextually concentrated in three main clusters: Indigenous evaluation scholarship from settler-colonial contexts, Afrocentric and African Indigenous evaluation paradigms, and globally oriented decolonial frameworks. The largest cluster of literature (n = 37) comes from settler-colonial contexts in Canada, Australia, Aotearoa/New Zealand, and the United States. This cluster share a common focus on addressing the ongoing legacies of colonialism in health, education, and research, and on creating evaluation practices that are culturally safe and responsive to the needs of Indigenous peoples [28,39,40]. Within this cluster, distinct regional paradigms are observed: in Aotearoa/New Zealand, Kaupapa Māori Evaluation (KME) provides a key theory-based practice rooted in Māori worldviews, rights to self-determination (Tino Rangatiratanga), and collective interdependence (whakapapa) [41,42]. North American scholarship emphasises Tribal Sovereignty and self-determination, developing frameworks such as the Indigenous Evaluation Framework (IEF) and approaches informed by Tribal Critical Race Theory (TCRT), which interrogate the intersections of race, colonisation, and sovereignty to advance culturally responsive and politically grounded evaluation [43,44].

The second cluster of scholarship (n = 10) is grounded in Indigenous African philosophies, promoting evaluation paradigms that are both contextually relevant and epistemologically just. Central to this work are concepts such as Ubuntu (*I am because we are*) emphasising interconnectedness and collective humanity, and Afrofeminism, which together inform frameworks such as Made in Africa Evaluation (MAE). These approaches seek to dismantle the imperial episteme and resist neocolonial logics in development and evaluation [26,27,45,46].

The third cluster includes records (n = 12) that took a global perspective or focused on other specific regions such as Asia and South America. These offered conceptual frameworks for decolonising research, ethics, and evaluation more generally, often within the fields of global health and international development [23,47] (S4 Table).

### Part II: Deconstruction VS. (re)construction

To organise the diverse literature included in this review, we developed a heuristic (Fig 2) that captures the nature of the literature as engaging in two concurrent, dialectical and complementary theoretical projects: 1) deconstruction (challenging colonial legacies) and 2) (re)construction of decolonial and Indigenous methodologies. Importantly, deconstruction and construction are not conceived as being confined to a single methodological approach in the included literature; rather, they span and underpin the literature variously across the spectrum (Fig 2). Nevertheless, they are understood and enacted differently within each type of methodological approach. For instance, the way colonial legacies are ‘deconstructed’ within an adaptive framework differs fundamentally in depth and intent from the structural dismantling found in more transformative frameworks outlined below. The typology therefore aims to illustrate not just where approaches sit on the spectrum, but how they uniquely operationalise the tasks of undoing colonial logic and constructing evaluative alternatives.

We begin firstly by examining the literature that focuses on the logic of ‘*deconstruction*’ (n = 17). This scholarship challenges the dominant logics of epistemic imperialism in evaluation, converging around key apparatuses. Critiques of the imperial episteme and false universalism highlight the transposition of ‘Eurocentric’ knowledge systems onto the rest of the world. For example, European humanism centres the rational, autonomous individual as the primary unit of agency and value. Decolonial scholars critique it for imposing a specific Western definition of ‘being human’ as one that separates the mind from the body and humanity from nature, as a universal standard, with evaluation itself implicated in this epistemological imperialism, as discussed by Dighe et al., (2023) [48] and Dutta et al. (2024) [49]. Historical ‘Eurocentrism’ is also challenged for privileging European history while relegating colonialism and Global South experiences to the margins, resulting in inadequate theoretical foundations for evaluation [48]. Puije and Satzinger (2025) extend this deconstructive project to the level of evaluation governance by historicising the Organisation for Economic Co-operation and Development (OECD) Development Assistance Committee criteria as a donor-centred apparatus that claims universality while structurally excluding those most affected, thereby encoding geopolitical power into what is presented as neutral technical judgement [50]. Similarly, van der Puije (2025) foregrounds the promotion of evidence as secular by showing how development evaluation’s modern epistemic commitments can render spiritual knowledge illegible, thereby reproducing epistemic exclusion even when participatory methods are used [51]. Some evaluation approaches also perpetuate reductive binaries, such as East/West or developed/developing, which homogenise colonised peoples and deny their agency and diversity. Additionally, the literature critiques authoritarian data production and neutrality by discussing how data practices reproduce racial hierarchies and systemic power imbalances, as argued by Zwiener-Collins et al. (2023) [52]. These hierarchies are maintained through extractive data practices, whereby information is effectively ‘mined’ or ‘extracted’ from communities as a raw commodity for the primary benefit of external researchers, institutions, or funding agencies [53]. This dynamic is widely described as helicopter science, in which external evaluators enter communities to collect data, depart with minimal local involvement in analysis, and offer little recognition or authorship to local evaluators, researchers or knowledge holders [44,54]. Such practices directly violate principles of Indigenous data sovereignty by denying communities authority over the governance, ownership, interpretation, and application of their own data. Another particularly salient example discussed in the literature is the persistent racial misclassification of Indigenous peoples in public health datasets, which scholars argue contributes to systematic erasure and what has been termed ‘data genocide’ [54], undermining both visibility and policy relevance for Indigenous communities.

Second, complementing this critique is a body of literature focused on ‘*(re)construction and Indigenisation*’, centring Indigenous worldviews, and reclaiming Indigenous ways of knowing, restoring lost identities, and defining value in culturally grounded terms and methods [55,56]. Central to this construction is a paradigmatic shift, where Indigenous-centred frameworks such as Kaupapa Māori, Made in Africa Evaluation (MAE), and the Indigenous Evaluation Framework (IEF) redefine value in terms of liberation, social justice, and community-determined measures of wellness, rather than against colonial benchmarks [22,46,48,57]. Equally important is the principle of epistemological equality, which calls for multi-epistemological partnerships that treat ‘non-Western’ relational ontologies and Indigenous knowledge systems not as alternatives but as equal knowledge traditions alongside ‘Eurocentric’ frameworks [26]. Methodologically, scholars advance alternative approaches that align with cultural protocols, employing proverbs, songs [45], relational accountability, non-extractive practices, 6 Rs (respect, relevance, reciprocity, responsibility, relationships, and relationality) [58], and integrative approaches such as Two-Eyed Seeing [59], an epistemological approach that encourages viewing the world through both Indigenous ways of knowing and ‘Eurocentric’ scientific knowledge simultaneously. A strong emphasis is placed on sovereignty and self-determination, operationalised through principles such as Tribal Sovereignty [60] and OCAP (Ownership, Control, Access, and Possession) to ensure that evaluation directly contributes to nation-building [56]. Finally, Dazzo (2025) pushes (re)construction by highlighting the potential for evaluation itself to become a practice of repair where validity is judged not only by coherence, but by whether evaluative work restores relationships, recognises harmed communities as epistemic authorities, and contributes to justice [61]. This reframing implies that reparative evaluation must involve redistributive commitments (of voice, time, and material support) so that participation does not simply supply data to unchanged extractive structures.

### Part III: Typology of decolonial approaches to evaluation

We organised the literature on decolonising and Indigenous evaluation into a typology defined by the level of transformation of epistemic power sought, the degree of Indigenous sovereignty asserted, and the centrality of Indigenous perspectives. This typology spans from approaches that pursue simple *adaptation* of existing evaluation systems to those that demand a *deeper transformative* philosophical and political reorientation (Fig 2). We present the domains in this order for narrative clarity, noting that this is non-linear structure and the domains are permeable and frequently overlap. Where a framework spanned more than one domain, we mapped it to each relevant domain and discuss the overlap explicitly. The concepts of deconstruction and (re)construction described above inform all of these approaches to a lesser or fuller extent and may overlap. For instance, participatory evaluation methods are discussed both as involving a distinct deconstruction methodological project and as having an integral component oriented toward (re)constructing participatory evaluation. In this case, this overlap is functional. Indigenous frameworks operationalise participatory methods (such as yarning circles) not just for data collection techniques, but as the necessary mechanisms to achieve the political goals of self-determination and relational accountability. Also, the literature reveals fluidity in how approaches in the typology are applied; some are conceptualised as overarching paradigms, while others are mobilised more as practical methodological approaches, particularly in relation to participatory and culturally responsive practices. The criteria distinguishing the domains are summarised in Table 2.

**Table 2:** Summary characteristics of the four domains of the typology.

	Indigenous evaluation methodologies	Decolonial and anticolonial methodologies	Participatory methodologies	Culturally sensitive methodologies
Philosophical/ epistemic orientation	Rooted in Indigenous ontologies, epistemologies and axiologies in which knowledge is relational, place-based and collectively held; value is defined through community-determined wellbeing rather than external benchmarks [21,62].	Critiques the coloniality of power and knowledge and treats evaluation as implicated in the coloniality of power and knowledge that outlives formal independence [63]. It pursues epistemic justice and structural transformation rather than accommodation [48].	Values local knowledge and stakeholder involvement, from instrumental to genuinely transformative participation	Seeks relevance and respect for cultural context while working within existing evaluation paradigms
Position on the spectrum of (deconstruction–(re)construction	Predominantly (re)constructive. It reclaims Indigenous ways of knowing and asserts sovereignty over the evaluation itself. Deconstruction is enacted implicitly through refusal of colonial benchmarks [20,34].	Strongly deconstructive. It historicises and dismantles colonial logics such as the imperial episteme and donor-centred OECD–DAC criteria [48,50], with (re)constructive intent that opens space for alternatives.	Limited deconstruction and partial (re)construction only where participation becomes community-led, otherwise co-produces within existing structures.	Engages neither task substantively and adapts methods within existing paradigms rather than undoing or rebuilding them.
Epistemic authority	Held by Indigenous communities and knowledge holders, who define value and govern the process	Shifts towards the colonised and their communities; evaluators accountable for redistributing epistemic power.	Shared in principle but often retained by external evaluators in practice; varies with depth of power-sharing.	Remains largely with external evaluators and commissioning institutions
Key methodological features	Centring community protocols, using yarning circles, relational practice and Indigenous data governance such as Ownership, Control, Access and Possession (OCAP)	Structural analyses of power and positionality, critique of dominant criteria, reflexive and emancipatory designs.	Coproduction, co-design, participatory appraisal, deliberative and empowerment methods, stakeholder-led data collection.	Cultural adaptation of instruments, culturally matched data collection, attention to language and context
Illustrative frameworks	Kaupapa Māori framework, and Indigenous Evaluation Framework [34,42].	Made in Africa Evaluation Framework [22,26,27].	Community-Based Participatory Research (CBPR); Participatory Rural Appraisal (PRA) [64].	Culturally Responsive Evaluation (CRE) and cultural competency approaches [65].
Intended application	Evaluation led by and for Indigenous communities in service of self-determination	Used to interrogate and transform the colonial structures of evaluation systems themselves	Used to involve communities and stakeholders, from consultation through to shared governance	Used to adapt existing tools to cultural context, improving relevance and acceptability

#### Indigenous evaluation methodologies.

In this domain, evaluation is anchored in the worldview, perspectives, values, philosophies, and lived experiences of specific Indigenous communities, honouring Indigenous sovereignty [33,60,66,67]. The researchers recognise Indigenous people as neither victims nor objects of research [68]. Methodology is intimately linked to assertions of self-determination and self-governance [34,56,69].

To honour these concepts, evaluation methodologies should be a holistic, relational, and contextualised experience of the place, time, community, and history [33], centred on Indigenous epistemologies and ways of knowing. Often driven by a decolonising intention [45], Bowman et al (2015) [60], LaFrance and Nichols (2008) [34], Polansky and Echo-Hawk (2021) [54], Goforth et al. (2022) [44] Rowe (2022) [56] and Eakins (2023) [68] highlight the Indigenous Evaluation Framework (IEF) that attempts to re-centre evaluation practices within Indigenous worldviews, lands, and cultures, governed by a set of interrelated values that collectively reframe evaluation as a place-based, relational, and sovereign practice. Central is the value “People of a Place”, which situates programmes and their evaluation within the specific historical, cultural, and spiritual geographies of communities [34]. The framework affirms ‘Individual sovereignty’ by rejecting comparison to standardised norms and instead adopting a holistic understanding of personal growth and worth. It centres community and family to frame evaluation as a collective, transparent practice oriented towards communal wellbeing rather than detached vertical public health metrics [34,70]. Sovereignty underpins these values by asserting Indigenous authority over data governance, intellectual property, and the use of evaluation for self-determined development and nation-building. Foundational to all is Indigenous knowledge, which legitimises diverse epistemologies, including empirical, intergenerational, and spiritual ways of knowing, as equally valid bases for evaluation design, interpretation, and use [34,68].

Kerr (2012) [41], Cram (2016) [42], and Cram et al. (2015) [62] discuss Kaupapa Māori Indigenous evaluation, where evaluation must be done *‘a Māori way*’ [67], using practices rooted in Tino Rangatiratanga (self-determination) and Whakapapa (genealogy) [42]. This defines value in terms of cultural integrity and resistance to capitalist exploitation. Similarly, Chilisa et al. (2016) [22], Dlakavu et al. (2022) [46], Easton (2012) [71], Boadu and Ile (2024) [72], and Kane and Archibald (2023) [45] build their work around an African relational paradigm where evaluations are rooted in philosophies such as Ubuntu (humanness, communitarianism, solidarity, interdependence), and uses local Indigenous Knowledge Systems (IKS), such as African proverbs [71] and oral traditions [45] as methods that *‘invite communities to dialogue with academics for decolonisation purposes,* elicit contextually grounded meanings of value and wellbeing, and centre community accountability. Kawakami et al’s. (2007) [33] work focused on Pacific/Kanaka Maoli, centring on relationality to land (‘āina) and family (‘ohana) and using narrative methods like mo‘olelo (storytelling). To implement decolonial evaluations, Indigenous methods such as storytelling/Mo’olelo [33], talking circles [60], culturally created manifestations (e.g., oli [chant], hula) [33], drawing on wisdom from ceremonies, language, elders, visions, and dreams [60], are used, as well as methods that centre Indigenous ways of knowing such as photovoice [57]. Johnson et al. (2025) cautions against attempts to operationalise Indigenous evaluation methods as the production of tools, indicators, or data collection plans [73]. Their account of kwayâtisiwin (“we prepare”) argues that evaluation-relevant work begins well before formal activities and includes ceremonial, relational, and embodied forms of preparation that are routinely rendered invisible by academic and funder evaluation logics. By insisting that Indigenous research proceeds on ‘Indian time’ and seasonal rhythms rather than quarter-by-quarter deadlines, they reframe the perceived universal timelines as a methodological constraint. Taken together, this domain represents the most transformative end of the spectrum because it emphasises Indigenous ownership, ways of doing and being. At its most radical, it asserts that Indigenous research and evaluation in Indigenous contexts should be conducted exclusively by Indigenous evaluators, who should be embedded in the evaluation context. Thereby, shifting epistemic power from external evaluators to Indigenous peoples.

#### Decolonial/anticolonial methodologies.

This domain, the second most transformative, includes literature that, similarly to the first approach, focuses on the deconstruction of colonial power, challenging ‘Eurocentric’ epistemologies, and examines the effects of imperialism, slavery, racial capitalism, racism, and colonialism on evaluation and methods. Dighe and Matthias (2024) [48], Chilisa and Mertens (2021) [26], and Smith (2012) [20] view evaluation as often functioning as an ‘instrument of epistemological imperialism’, where ‘non-Western’ ontologies should rather be acknowledged as equal (not a lesser alternative). Dutta et al. (2024) [49] discuss racial capitalism as an oppressive structural framework that positions economic exploitation and profit goals at the intersection of race, colonialism, and whiteness. The concept identifies Slavery/Capitalism as a foundational pillar of white supremacy, where historically blackness was equated with property and commodity, a form of exploitation in a wider neoliberal model of labour exploitation. It perpetuates the colonial-capitalist formation, ensuring that capitalist expansion relies upon racialised hierarchies to extract value from the labour, land, and lives of marginalised peoples. Evaluations in these systems focus on individual behaviours, sidelining the structural accounts of colonialism, imperialism, and racial capitalism that constitute poor health outcomes at the racialised, classed, and gendered margins. Pant et al. (2022) [23], Goldman et al. (2024) [32], Dutta et al. (2024) [49], Backhouse (2023) [74], Melro et al. (2023) [28], and Cram et al. (2015) [62], however, frame decolonial/anti colonial evaluation as a means to challenge neoliberal economic systems which support continued colonisation and colonial projects, restructure power imbalances, and identify the structural causes of inequality rather than assigning individual blame. Thus, decolonisation is here understood as the restructuring of power relations in global evaluation knowledge production [22], as an intentional process of undoing, unlearning, and dismantling unjust practices [46,74].

The literature in this domain focuses on the ethical and methodological shift away from extractive research [58] and moving away from characteristics like ‘either/or’ thinking, perfectionism, and the ‘worship of the written word’ [32]. Here, key methods and tools emphasise evaluation practices grounded in local cultures and modes of knowledge-sharing. These include the use of local languages [22], storytelling, and participatory videos [74], alongside established qualitative and participatory approaches such as participatory ethnography, Most Significant Change (MSC), participatory rural appraisal, participatory narrative inquiry, and sense-making - a method that engages evaluation stakeholders in collaboratively interpreting and making meaning of data to guide decision-making [46]. Zwiener-Collins et al. (2023) [52] advance the field by interrogating the Enlightenment paradigm in quantitative methods, examining how standard statistical concepts, such as Gross Domestic Product (GDP) and the Human Development Index (HDI), can reproduce power hierarchies. However, the use of indigenised ‘traditional’ approaches to evaluation like Randomised Clinical Trials (RCTs) can be acceptable, as discussed by Chilisa and Mertens (2021) [26].

To summarise, while Indigenous and decolonial domains both challenge colonial power, decolonial/anticolonial methodologies and attempt to deconstruct ‘Eurocentric’ epistemologies, expose structural inequities and embed Indigenous worldviews in evaluation practice, Indigenous evaluation methodologies go further along the spectrum by, asserting Indigenous ownership, and actively reconstructing power relations to restore epistemic and political agency to Indigenous communities.

#### Participatory methodologies.

The literature in this domain is centred on meaningful community participation, inclusion in research processes and methodological adaptation to enhance local validity and utility, prioritising collaboration, relational ethics, and capacity building. Therefore, the methods here are designed to facilitate dialogue, knowledge co-production, and relational accountability, but do not necessarily seek to fundamentally challenge or redistribute unfair structures of epistemic or political power, distinguishing them from the more transformative decolonial and Indigenous evaluation approaches evaluation approaches discussed above. However, Melro et al. (2023) [28], Renmans et al. (2022) [47], Kelly and Htwe (2024) [53], and Povey et al. (2023) [39] highlight the need to consider evaluator’s positionality and Indigenous worldviews, and commitment to actively build a climate of trust and respect, ensuring stakeholders have meaningful and have meaningful roles and share power in the process [60,65], honouring the strengths and diversity within communities and designing evaluations to be responsive to the cultural context(s) under examination. This literature advocates for an approach where the evaluator and stakeholders jointly make decisions about evaluation questions, conduct, and how results are shared and disseminated [75]. It moves away from the received view of the evaluator as a detached ‘objective’ observer focusing on what is ‘objectively’ measurable and potentially excluding Indigenous knowledge in the development of evaluative measures. This may result in the exclusion of knowledge such as traditional knowledge (i.e., wisdom passed down from ancestors), revealed knowledge (i.e., knowledge gained through dreams or ceremony), or feelings about community wellbeing [68]. In complex systems, Bailie et al. (2020) [76] recommend continuous reflection, learning, and co-production to design and adapt interventions effectively.

Some of the literature from Africa, relational-based evaluations reflect the African logic of circularity, emphasising that relationships and sub-relationships concurrently inform one another. The processes are cyclical, evolving, and generative, reflecting that reality is not static or material but includes spiritual and non-material existence, necessitating continuous feedback and adaptation [22]. This contrasts with the characteristics of traditional ‘Eurocentric’ linear logic, which often follows a rigid, step-by-step process (e.g., from inputs to outcomes to impact). Aman et al. [64] discuss the level of engagement for communities in evaluation and research as existing on a continuum, ranging from minimal involvement to full control, encompassing roles such as advisor, collaborator, and leader. At the low end, the community acts as an advisor, providing input on strategies and tools but holding limited decision-making control. Moving along the spectrum, the community becomes a collaborator, actively involved in shared decision-making, co-creating tools, and participating in data collection and interpretation. At the high end of the continuum, often described as Community-Based Participatory Research (CBPR), the community functions as the leader; they drive the entire project, defining the research questions, setting timelines, making staffing decisions, and having full ownership over the evaluation.

The literature highlights the use of some methods that are commonly used in traditional evaluations, such as storytelling, participatory social mapping, community meetings, focus groups, and interviews, Participatory Rural Appraisal (PRA) [28,46,75], and some more context/culturally-specific methods, such as the use of participatory design principles and the Aboriginal and Torres Strait Islander quality appraisal tool to assess quality of care, as developed by Povey et al. (2023) [39]. The use of ‘Talking Circles’ or ‘Yarning Circles’ as a culturally responsive practice are perceived to help capture community attitudes and wellbeing and foster a space for shared understanding [70,77]. The emphasis is on a collaborative journey where the evaluator acts as a facilitator rather than an ‘independent’ expert, and where community capacity is strengthened through the process itself.

#### Culturally sensitive approaches.

This domain is largely defined by what the decolonial and Indigenous literature seeks to move beyond. It includes approaches that prioritise accommodating cultural differences or reducing offending racialised and ethnic minorities, without fundamentally challenging ‘Eurocentric’ epistemological dominance or conceding control to Indigenous groups, such as ensuring basic language translation or cultural politeness, without altering who defines success or controls the process, as discussed by Kelly and Htwe (2024) [53].

Much of the literature included in this review actively critiques the approaches that stop at this level, often contrasting them with the deeper transformation found in more participatory, decolonial and Indigenous evaluation methodologies. For example, the notion of cultural competency (knowledge of a culture) was perceived as passive in the literature and authors instead advocate for the active requirement of cultural safety, which, as outlined above, demands that evaluators critically reflect on their own power and positionality to ensure their practice does not harm participants [23,28].

Cultural safety, or Kawa whakaruruhau in Māori, is a methodological and ethical requirement that shifts the focus from cultural awareness to recognising how Indigenous peoples are situated within structural inequalities, as argued by Cargo et al. [40]. In this sense, cultural safety is distinct from Indigenous approaches in that it is often the mechanism through which non-Indigenous evaluators can ethically engage with Indigenous worldviews. In practice, it requires critical self-reflection on evaluator positionality, avoidance of stereotypes, and acknowledgement of how dominant cultural identities, particularly whiteness, shape power relations [40,42,60]. Methodologically, it requires privileging Indigenous institutions, honouring cultural protocols, and upholding relational accountability, ensuring that evaluations are guided by Elders and communities and that authority rests with Indigenous groups. As Brown and Di Lallo [78] and Hood (2015) [65] argue, validity rests on the quality of relationships embedded in the process, which necessitates time, trust-building, and shared understanding during the early planning and design stages. Honouring protocols, or Kia tūpato (being careful), further requires evaluators to act with political astuteness, cultural reflexivity, and alignment with tikanga (cultural rules and responsibilities), often supported by the mentorship of Elders or cultural practitioners, as argued by Cram (2016) [42]. In clinical settings, Sehgal et al. (2024) developed a culturally safe patient complexity assessment framework for urban Indigenous primary care. They used a modified e-Delphi process to specify nine domains and 27 concepts, explicitly incorporating factors that conventional biomedical complexity tools tend to marginalise, such as adverse life experiences (including residential school), access to resources, and healthcare violence, alongside culture, resilience, and non-biomedical beliefs about health and illness [79]. Finally, Thiessen and Chouinard suggest that cultural adaptation does not, by itself, guarantee safety [80]. Practice-based reflections from a culturally responsive, Indigenous-focused evaluation describe how participants’ trauma surfaced vividly during data collection, prompting evaluators to question whether their methods risked re-traumatisation and to argue that trauma-informed practice should be treated as necessary rather than optional. This extends the critique of ‘minimal adaptation culturally sensitive approaches’ (that do not explicitly anticipate trauma, emotional labour, and culturally grounded practices of care) and may inadvertently reproduce extractive dynamics, even when participatory language and culturally appropriate approaches are used. As such, lack of trauma-informed approaches and surface-level adaptation are described as an immature form of decolonisation praxis [53].

### Part IV: Contested questions

Our synthesis of the literature reveals several key sites of debate and contestation that are central to the future of decolonial and Indigenous evaluation. The most consistently debated question in the literature concerns the role and identity of the evaluator. The discussion reorients discourse from an insider/outsider binary to a more nuanced interrogation of power, legitimacy, and accountability. On the one hand, the principle of self-determination, central to Indigenous sovereignty paradigms, strongly implies that communities should lead their own evaluations, positioning insiders not just as participants but as the primary knowers [27,43]. On the other hand, the literature acknowledges the reality of evaluations being conducted by external evaluators and grapples with how this can be done ethically. The consensus seems to be that this requires a shift in the evaluator’s role from a detached, objective expert to that of a humble facilitator and accountable ally [74]. This has led to a critical focus on the evaluator’s positionality, as reflected in the discussion of the approaches above. As Renmans et al. (2022) [47] argue, researchers external to the evaluation context must recognise that their knowledge production is constrained by their social and cultural position and actively engage with the power inequities inherent to their role. Another issue is the separation between implementation, impact evaluation and process evaluation, with some authors noting how rigid walls between implementers and evaluators can marginalise local knowledge and reduce the utility of evaluation findings [34]. Indigenous and participatory frameworks, by contrast, emphasise interlaced practice, where evaluators and implementors work and learn together, and the process itself is valued as a source of knowledge [34,68], treating learning as continuous rather than one-time reflection, recognising that understanding how and why changes occur is as important as measuring final evaluation outcomes. The literature suggests the defining question for the evaluator(s) positionality is not ‘who’ you are, but ‘to whom you are accountable’ [40]. This reframes the debate from one of identity to one of political commitment, solidarity and relational accountability, challenging the professional norms that often privilege technical credentials over relational skills and demonstrated trustworthiness.

Second is the question of how to engage with multiple epistemologies, particularly the integration of decolonial and ‘Eurocentric’ epistemologies and methodologies. We have not identified a clear answer within the literature. However, some approaches, like Two-Eyed Seeing, propose a path of weaving together the strengths of both knowledge systems [64]. As Dighe et al. (2023) [48] argue, a core tenet of decolonisation is to recognise ‘non-Western’ ontological and epistemological frames as ‘equal’, rather than ‘alternative’, but providing distinct insights. Others assert the need to create and hold sovereign spaces for Indigenous knowledge systems. For example, proponents of frameworks like Kaupapa Māori Evaluation and the Indigenous Evaluation Framework (IEF) assert that Indigenous knowledge systems and worldviews are complete and legitimate in their own right; they do not require validation by, or integration with, ‘Eurocentric’ frameworks [41,42]. The tension, therefore, is between a pragmatic desire for dialogue and integration, and a political and philosophical assertion of epistemological sovereignty. This is an important debate, as the path of integration carries the risk of co-optation of decolonial approaches and the subtle recentring of ‘Eurocentric’ norms, while a sovereigntist approach can face challenges of marginalisation within mainstream funding and policy environments.

The final and most pragmatic contested question is around the applicability and implementation of these frameworks within the dominant, often neo-colonial, structures of global health evaluation. Our synthesis of the included literature, like that of Pant et al. (2022) [23] finds a ‘praxis gap’ between the radical theoretical aspirations of the literature and the scarcity of empirical studies demonstrating their application and effectiveness at scale. The literature questions the feasibility of embedding these relational, emergent, and community-led processes into the rigid, bureaucratic project cycles of funders and governments [32], unless their value, appropriate use, and the risks of instrumentalisation or tokenistic application are better documented and analysed.

## Discussion

This scoping review maps a rapidly evolving yet conceptually fragmented field that aims to decolonise and Indigenise evaluation methodology and practice within global health. Our synthesis demonstrates that the movement to decolonise and Indigenise evaluation is a project engaged in two concurrent and complementary projects: the deconstruction of colonial systems and the construction of decolonial Indigenous-centred alternatives.

A key strength of this study lies in its advancement of conceptual synthesis in a field where the understanding of theoretical framing and implications for practice has been patchy and dependent on concepts developed in other disciplines, such as political theory, international development, education, and psychology with limited novel conceptualisation in global health. While our findings document the plurality of theoretical and conceptual frameworks, there are few resources on reporting on the operationalisation of these and their use in real-world evaluations may currently be limited. Melro et al. (2023) [28] engage directly with this tension by examining which theoretical frameworks are used and implemented, and which evaluation approaches, methods, tools, and frameworks are applied to assess effectiveness across settler-colonial contexts. In their scoping review, they found that among the evaluations of 14 health professional education interventions, only two used a theoretical evaluation framework- both grounded in critical or cultural consciousness. Our review expands the interrogation of some of the questions Melro and colleagues [28] raised by offering a structured typology that describes theoretical and methodological developments within an evolving decolonial paradigm. In doing so, it contributes to strengthening interdisciplinary dialogue by integrating critical literature on decolonising evaluation methodology from political science, social justice, and international development disciplines into the global health evaluation communities.

Our typology illustrates how scholarship in decolonial and Indigenous evaluation methodologies spans multi-layered intellectual and political projects, presenting a spectrum of approaches ranging from minimal adaptation, as observed in culturally sensitive methodologies, to profound philosophical and liberational restructuring of colonial, oppressive and capitalist systems of evaluation and knowledge production.

Within this spectrum, methodologies variously described as Indigenous, decolonial, participatory, or culturally responsive, articulate distinct yet overlapping commitments to equity, self-determination, and epistemic plurality. This wide spectrum of methodologies is often conflated or used interchangeably in the literature. A key strength of this review lies in its attempt to bring conceptual and analytical clarity to this evolving landscape through the development of a typology that distinguishes between levels and forms of decolonial transformation in evaluation methodology. While recognising that these approaches are neither linear (developed one after the other) nor rigid, and that some aspects traverse multiple approaches/domains, our synthesis contributes to advancing definitional precision in an area often marked by conceptual ambiguity. We therefore call for greater precision in the use of terminology in the field to improve transparency in the description of the approach being taken. The increasing use of ‘decolonial’ as a rhetorical or fashionable label for work that is participatory or culturally adaptive - without engaging in the deeper structural, epistemic, and political transformations central to decolonial evaluation - risks diluting the concept’s meaning and intent [72,81]. While we acknowledge that this challenge reflects a broader issue across evaluation practice, it is particularly important to highlight it within this emerging field to preserve the analytical and transformative integrity, rigour and meaningfulness of decolonial scholarship.

Concretely, symbolic adoption tends to retain external control while changing only vocabulary by relabelling a standard satisfaction survey or stakeholder consultation as participatory or decolonial, while communities and local actors still have limited power in setting the research agenda, owning the data and defining success. In their account of community-development evaluation in Rakhine State, Myanmar, Kelly and Htwe (2024) describe measures such as language translation and cultural courtesy that are relabelled as decolonising, yet leaving untouched who defines success and controls the process, characterising such surface-level adaptation as an ‘immature’ form of decolonisation praxis [53]. Substantive change instead reworks the criteria of value and who sets them. Sehgal et al.’s (2024) culturally safe complexity assessment for urban Indigenous primary care used a modified e-Delphi with Indigenous experts to embed factors that conventional biomedical tools marginalise — including residential (boarding) school experience, access to resources and experiences of healthcare violence — thereby shifting what the evaluation treats as legitimate evidence [79]. Our typology contributes to this conversation by giving evaluation-specific definitional structure to a field that has largely borrowed its terms from adjacent disciplines.

### A note on the practice of decolonial inquiry

The place where this review was conducted matters. The neoliberal structure of academia forces academics to continually demonstrate individual growth, often measured by securing grants, developing new applications of knowledge, and showcasing impact both within and outside academic circles, to maintain employment. The commodification of knowledge, along with the way it tends to be framed in academic settings, as something to be acquired for personal benefit and in the name of progress, has contributed to the perpetuation of epistemic violence. Moreover, the neoliberal structure of academia increasingly intensifies knowledge production (more grants, more papers, more ‘impact’) and, compounded by increasing job insecurity. While these structures often emphasise the importance of interventions aimed at producing outputs, they rarely allow space for simply existing with knowledge. This lack of space leads to the constant appropriation of knowledge into familiar frameworks and canons, rather than creating room for alternative ways of understanding and being. Reardon and TallBear (2012) [82] observe how, even though contemporary scientists may reject overt racism, the entanglement of ‘Whiteness’, ownership, and the human sciences continues to replicate colonial power structures and hierarchies [83,84]. This leads us to a position, along with global health voices [83,84], where we question the ability of global health institutions to dismantle power imbalances entirely and redefine the field itself.

From the outset of this project, we have felt increasing discomfort with the rigidity inherent to scoping review methodology, acknowledging they risk simply reflecting the same rigidity and hierarchies in the discipline of scoping reviews, including the arbitrary boundaries, normative biases, hierarchical ordering, and ‘Eurocentric’ foundations that typologies and categorisations often impose. Chambers et al. (2018) [25], highlight the mismatch between conventional scoping review methods, rooted in linear, reductionist, and ‘Eurocentric’ practices such as evidence hierarchies, and the holistic, relational, and critical orientations of Indigenous knowledge systems. We too questioned the appropriateness of scoping review methodologies and our approach to synthesis for the literature that we were reviewing. We also understand the tension afforded by an evolving canon of appropriate terminology related to this endeavour. In particular, the characterisation of dominant and non-dominant entities as, for example ‘Eurocentric’ or ‘Global North’ and ‘Global South’ or ‘Indigenous’ have fallen out of favour in some arenas. We view these nonetheless as still of value for search and reporting methods to reflect and capture changing thinking over time, but equally acknowledge they risk simply reflecting the same rigidity and hierarchies in the discipline of scoping reviews and global health.

The initial step in a review, which involves establishing conceptual boundaries around the inclusion of literature, raised questions for us about whether and how such boundaries could be defined, especially given our limited understanding of Indigenous knowledge systems. The act of delineating and re-delineating borders has historically been a colonial exercise, creating artificial divisions within communities and fuelling conflicts that continue today. Similarly, categorising and segmenting knowledge, as we have, into different domains may result in contrived separations that risk misrepresenting interconnected systems of understanding. While in the context of a review this process might foster understanding and creativity, it could also lead to unintended associations or interpretations contrary to the original intentions of the knowledge creators. The practice of extracting data also evokes colonial practices of exploiting natural resources, land, and people from colonised regions. Colonisers not only imposed their knowledge systems on the colonised but also extracted and appropriated Indigenous knowledge without adequate recognition or attribution. Couldry and Mejias (2019) [85] have pointed out that while historical colonialism focused on land, resources, and people, contemporary forms of colonialism extend this practice by commodifying human life through the extraction of value from data. Its intention – to extract, synthesise data and gain mastery over knowledge - reflects a broader legacy of the ‘coloniality of knowledge’ [19,63].

Ocean Vuong, a Vietnamese American writer, reflects on the violence embedded in the English language, evident in celebratory expressions such as ‘killing it’ or ‘smashing it’ [86] and considers how such metaphors shape how people perceive themselves and relate to others. Similarly, we are attentive to the violent lexicon of scoping reviews, terms like extraction or exclusion, and to the risks that these practices may lead to commodifying language appropriating Indigenous knowledge.

Finally, when considering decolonial scholarship on global health evaluations, it is necessary to acknowledge the discomfort that these critiques provoke. The discomfort inherent in challenging methods central to “Eurocentric” epistemology perhaps helps explain the slow uptake of decolonial evaluation methodology scholarship. Looking ahead, such hesitations and forms of resistance may continue to shape this field and practice.

### Implications for research and practice

Typically, a scoping review concludes by outlining a future research agenda based on the identified gaps. However, to do so without critical reflection would be to replicate the very epistemic authority highlighted by this review. The power to define and lead the future evaluation research agendas rightfully belongs to communities themselves, as part of practising Indigenous sovereignty. Those currently using/benefiting from entrenched unfair power structures also need to be willing to be challenged to change their practice and relinquish their control of the evaluation agenda. As the literature argues, a core tenet of decolonisation is the assertion of self-determination, which necessarily includes control over knowledge production and the questions that are asked, as discussed by Smith (2012) [20] and Bowman-Farrell (2018) [43]. Therefore, what follows is offered as potential areas for consideration, to be taken up, adapted, or rightly ignored by those who are leading work on the ground.

Future research is needed to document how decolonial and Indigenous evaluation frameworks are implemented and how their effectiveness is measured in real-world settings, the institutional barriers encountered, and the strategies used to navigate them, thereby bridging the disjuncture between theory and documented practice. Furthermore, the literature needs to advance beyond using Indigenous theory primarily to guide early evaluation design to providing robust guidance on how frameworks like the IEF, Kaupapa Māori, or MAE can inform data analysis, indicator selection, and the interpretation of findings. This would involve developing methodologies to measure community-defined concepts of, for example, transformation, wellness, and social justice, rather than simply adapting conventional colonial benchmarks of success. Finally, an area for future inquiry is the interface between decolonial methodologies and the institutional structures of commissioning, exploring how the rigid, linear, and accountability-driven demands of donors and governments can be reconciled with the emergent, relational, and community-led principles of decolonial evaluation.

Responsibility for acting on these implications is, moreover, not evenly distributed, and it should be proportional to power. Much of the decolonising literature addresses its recommendations to individual researchers, yet the labour and moral weight of this work tend to fall on those least able to carry this. The task of redressing institutional racism is offloaded onto racially minoritised and Global South scholars as unrecognised ‘diversity work’, even as they are penalised for the dissent it requires [87]. Early career researchers, rarely powerful and often precarious, are expected to model decolonial practice in institutions with little appetite for the redistribution real change demands [88]. The heaviest obligations, however, should rest with those who benefit most from current arrangements and who control the resources, standards and careers that sustain them, namely funders, commissioners, senior leadership in Global North institutions — some of whom have begun to act, as with the Australian Productivity Commission’s Indigenous Evaluation Strategy [75]. We set out implications for policy and practice by actor in Table 3 and expand on them in our reflexive planning guide for evaluators [38].

**Table 3:** Implications for policy and practice by actor.

ACTOR	IMPLICATIONS
Funders and commissioners	• Project and reporting cycles need de-standardising to allow relational, emergent and flexible timelines, accommodating trust building and community-led work. • Community data-governance arrangements require explicit resourcing. • Proposals warrant assessment and reward for equitable, decolonial practices. • Evaluation capacity within communities, not only within external institutions, needs de-risking and funding.
Policymakers	• Commissioning standards and national evaluation policy need to embed decolonial and Indigenous approaches. • Evaluative authority and data governance belong, at least in part, with the communities concerned.
Global Health Educators	• Global health evaluation curricula need to recognise and teach decolonial and Indigenous methodologies. • Students require equipping to interrogate positionality, power, and the colonial legacies of evaluation. • Indigenous-led frameworks and scholarship belong in the centre of curriculum, not at its margins.
Practitioners and evaluators	• Consider whether you and your team are best placed to lead the evaluation, who is best placed to lead, and how embedded you are in the community and programme, warrants honest scrutiny. • Local epistemologies merit exploration and, where appropriate, adoption as guiding frameworks. • Evaluation language needs scrutiny, and Indigenous methods should not be co-opted (e.g., renaming a focus group a ‘story circle’). • Indicators and success are best co-defined with communities, with consent and data governance (e.g., OCAP) treated as relational, not procedural. • Positionality and accountability belong explicitly in evaluation plans, with authorship agreed before fieldwork and findings co-interpreted and returned locally. • Accounts of the evaluation should record which decolonial or Indigenous principles were achieved and which were not.

## Conclusion

This scoping review demonstrates that the movement to decolonise and Indigenise evaluation is not a monolithic project but a multi-layered intellectual and political endeavour that fundamentally seeks to reimagine the field’s purpose, ethics, and practice. We situate our findings within wider debates on social justice and epistemic freedom. By bringing conceptual clarity through our typology, we address the critical need for analytical integrity in a field where ‘decolonial’ risks becoming a rhetorical label for work that is participatory, thereby diluting its transformative potential. The path forward, therefore, requires a dual commitment. First, evaluators and practitioners must move to consider ways to reframe their work through decolonial and/or Indigenous lens, and educators should include teaching of these approaches in global health evaluation curriculum. Concurrently, and just as critically, funders and commissioning bodies must create the necessary institutional space for this work to flourish. This involves reforming the rigid, neo-colonial structures of accountability- from procurement processes to reporting requirements- that currently inhibit the emergent and relational principles of decolonial evaluation. Ultimately, the future of a just evaluation practice hinges on the collective ability of all actors in the evaluation ecosystem to bridge this gap between transformative ideals and institutional realities.

## Supporting information

S1 Table The search terms used in the scoping review. (DOCX)

S2 Table Grey literature sources. List of organisational websites and repositories searched, with URLs.
(DOCX)

S3 Table PRISMA-ScR checklist. (DOCX)

S4 Table Characteristics of included studies. Data charting table for all 59 included records.
(DOCX)
