# Supplementary material for: Decolonising global health evaluations: A scoping review of methodological frameworks
Source: PLOS Glob Public Health. 2026 Sep 25;6(9):e0007362. doi: 10.1371/journal.pgph.0007362 (PMC13614595; doi:10.1371/journal.pgph.0007362)
Supplement: Supplementary file 1 [file pgph.0007362.s005.pdf]

LONDON SCHOOL of HYGIENE &TROPICAL MEDICINE

18 March 2026

Dr Dhan Raven San Juan Sanguenza,
*PLOS Global Public Health*

Dr Dhan Raven San Juan Sanguenza,

We are grateful for the opportunity to resubmit our manuscript 'Decolonising Global Health Evaluations: A Scoping Review of Methodological Frameworks' following the editorial correspondence regarding our original submission (PGPH-D-26-00281). We write to detail the steps taken to address the comments from the editors in this revised submission.

The decision on our first submission was on the grounds that the literature search had been completed over a year prior to submission and therefore did not provide a sufficiently up-to-date overview of the field. No external peer review was conducted, and no reviewer comments were received. We subsequently appealed this decision, and following careful consideration, the Editorial team (Jan Louis Adajo Non) upheld the decision, and reiterated that a revised manuscript with an updated literature search would be welcome as a new submission. We have now done so.

**Editorial concern: Literature search not sufficiently up to date**

We have fully addressed this concern. On 12 February 2026, we rerun the search using the same databases (Medline Ovid, Global Health, Embase), search strings, and grey literature sources as the first round of search. This ensured that the updated search was directly comparable to the original and that no methodological differences were introduced between the two rounds. The updated search identified additional records meeting the inclusion criteria bringing the total included records to 59. These new records have been analysed and integrated into the manuscript. Specifically, the following revisions were made:

1. The abstract has been updated to reflect the revised search numbers.
2. The introduction has been revised to more explicitly position this manuscript's contribution relative to the two existing scoping reviews in this space (Pant et al. (2022) and Stefanski et al. (2025)) clarifying how our analytical typology and global scope advance beyond these prior syntheses.
3. The methods sections, including describing the updated search process.
4. The PRISMA flow diagram (Figure 1) has been revised to reflect the updated search numbers, including the combined identification, screening, and inclusion figures from both search rounds.

5. The characteristics of the included studies has been updated to reflect the trends in literature, showing that 53% of all included records (n=31) were published between 2022 and 2025, reinforcing the timeliness and relevance of this synthesis.
6. The results section has been updated to incorporate the 10 new records, including findings of newly identified papers within the relevant domains of the typology.

The new records did not require revision to the analytical framework, typology, or conclusions, rather, they provided additional depth, and examples for the already developed analysis, strengthening the overall synthesis

**Editorial comment: PRISMA-ScR checklist**

The completed PRISMA Extension for Scoping Reviews (PRISMA-ScR) checklist has been uploaded as a separate supporting information file as requested. We note that the checklist was also included as Appendix C within the Supporting Information document accompanying the manuscript.

We believe this revised submission addresses the editorial concerns on our previous submission. We hope the updated manuscript is now meeting the expectations of PLOS Global Public Health, and we remain available to provide any further information the editorial team may require.

Yours sincerely,

Sali Hafez

DrPH candidate
London School of Hygiene and Tropical Medicine
