# Supplementary material for: Decolonising global health evaluations: A scoping review of methodological frameworks
Source: PLOS Glob Public Health. 2026 Sep 25;6(9):e0007362. doi: 10.1371/journal.pgph.0007362 (PMC13614595; doi:10.1371/journal.pgph.0007362)
Supplement: Supplementary file 2 [file pgph.0007362.s001.docx]

S1_Table search terms

| Area 1: Evaluations | Area 2: Global Health and International Development | Area 3: Decolonising | Area 4: Methods |
| --- | --- | --- | --- |
| “Evaluation”  “Evaluating”  “Assessing”  “Assessment”  “Impact” | “Global Health”  “Global Public Health”  “International Health”  “Public Health”  “Health”  “Healthcare”  “International Development”  “Development”  “Aid” | “Decolonising”  “Decolonial”  “anti-colonial”  “Indigenous”  “Aboriginal”  “Traditional”  “Equity”  “Equitable” | “Methods”  “Framework”  “Conceptual framework”  “Concept’  “Guide”  “Guidance”  “Tool”  “Theory”  “Principle” |
