# Supplementary material for: Decolonising global health evaluations: A scoping review of methodological frameworks
Source: PLOS Glob Public Health. 2026 Sep 25;6(9):e0007362. doi: 10.1371/journal.pgph.0007362 (PMC13614595; doi:10.1371/journal.pgph.0007362)
Supplement: Supplementary file 3 [file pgph.0007362.s002.docx]

S2_Table Supporting Information: Grey Literature Websites

| Title | Website |
| --- | --- |
| Google | https://www.google.com/?client=safari |
| Google Scholar | https://scholar.google.com |
| Australian Evaluation Society | https://www.aes.asn.au |
| Canadian Evaluation Society | https://evaluationcanada.ca |
| American Evaluation Society | https://www.eval.org |
| European Evaluation Society | https://europeanevaluation.org |
| African Evaluation Association | https://afrea.org |
| Asia Pacific Evaluation Association | https://asiapacificeval.org |
| Better Evaluation | https://www.betterevaluation.org |
| EvalIndigenous | https://www.evalindigenous.net |
| Indigenous Evaluation Network | https://www.indigenouseval.org |
| Carumba Institute | https://www.qut.edu.au/research/carumba-institute |
| Maamwizing Indigenous Research Institute | https://laurentian.ca/research/centres/maamwizing |
| Ānako Indigenous Research Institute | https://carleton.ca/anako/ |
| Relief Web | https://reliefweb.int |
| Doctors Without Borders (Resource Centre) | https://www.msf.org/resource-centre |
| World Health Organization (Resources) | https://www.who.int/resources |
