# Supplementary material for: Decolonising global health evaluations: A scoping review of methodological frameworks
Source: PLOS Glob Public Health. 2026 Sep 25;6(9):e0007362. doi: 10.1371/journal.pgph.0007362 (PMC13614595; doi:10.1371/journal.pgph.0007362)
Supplement: Supplementary file 5 [file pgph.0007362.s004.docx]

S4_Table Supporting Information Characteristics of Included Studies (n=59)

| *Paper* | *Evaluation focus and context* | *Summary* | *Featured Framework(s)* | *Theoretical Underpinning* | *Approaches* |
| --- | --- | --- | --- | --- | --- |
| Johnson et al (2025) [1]Kwayâtisiwin (“We Prepare”): Understanding the Methods of Preparation for Evaluating Nêhiyawak-Based Research (Peer-reviewed paper) | Evaluation preparation in Nêhiyawak research in the context of Canada | The paper discusses evaluation preparation as relational, land-based, and ceremonial practice. It the locates “preparation” as a form of accountability: a way of becoming able to evaluate in good relation, rather than extracting judgements. They describe three interconnected methods of preparation: Matotisiw (sweat lodge ceremony), Mawisowin (berry picking), and Kaskikwâsowin (sewing ribbon skirts), presented as practices through which evaluators learn, repair, and situate themselves. | Preparation as an evaluation method (Matotisiw; Mawisowin; Kaskikwâsowin) | Indigenous ontologies and relational epistemologies. It also draws on decolonial refusal of extractive timelines and land-based and ceremonial grounding of inquiry as legitimacy. | Reflexive, narrative/interpretive exposition organised through the three preparation practices; self-location and relational learning are treated as the core methodological moves (rather than a stepwise instrument/tool). |
| Seneca et al. (2025) [2]Responsibility to All Relations: Indigenous Evaluation of an AI/AN Public Health Workforce Development Program (Peer-reviewed paper) | Evaluation of Urban Indian Health Institute’s Public Health Training Program (PHTP), focused on strengthening the urban American Indian/Alaska Native public health workforce in the context of United States | The paper describe a mixed evidence strategy intended to centre trainee voice and relational meaning: trainee biographies, art statements, and a trainee story, alongside more conventional evaluation elements (e.g., indicators). | Indigenous Evaluation Framework | Indigenous evaluation paradigms foregrounding relational accountability, community benefit, and decolonising data; validity grounded in Indigenous values and community-defined success. | Community-led evaluation using mixed-methods, arts- and story-based evidence (biographies, art statements, narrative (trainee story) plus indicator-linked assessment aligned to IEF values and programme indicators. |
| Thiessen and Chouinard (2025) [3]Recognizing the Need for a Trauma-Informed Approach in the Evaluation of an Indigenous Program (Peer-reviewed paper) | Evaluation of Indigenous program within non-Indigenous institution in the settler colonial context of Canada. | The paper discusses the need for trauma-informed approaches to prevent extractive and harmful evaluation encounters. The authors distinguish multiple trauma-relevant layers: historical/collective trauma that shapes programme contexts; trauma that may be reactivated through evaluation encounters (questions, interviews, reporting); and trauma experienced by evaluators. They propose trauma-informed evaluation as an ethical and methodological necessity and mobilise established trauma-informed principles to translate this into evaluation practice. | Culturally responsive evaluation extended through trauma-informed approach (including 4 Rs and 6 Rs) as a reflective guide for evaluation relationships and responsibilities. | Culturally responsive evaluation and Indigenous evaluation ethics (relational accountability, minimising harm, attention to power and historical violence). | Trauma-informed interviewing; relational engagement |
| Dazzo (2025). [4]Evaluation as Reparation, Reclamation, and Renewal: Reorienting Our Justice Signposts Toward Restorative Evaluation (Peer-reviewed paper) | Reflexive evaluation theory paper using a transformative participatory evaluation example with the Forensic Anthropology Foundation and Kaqchikel Maya community members in Guatemala. | The paper reframes evaluation as restorative and reparative practice oriented toward justice and healing. The paper describes how donor-mandated measures were de-centred in practice, while narrative evidence (including oral tradition) and Maya cosmovision were foregrounded to shape questions, interpretations, and funder-facing accounts. The signposts offered reparation, reclamation, renewal function as orienting commitments for evaluators to move beyond ‘do no harm’ toward explicit redistributive and reparative action (including compensation and material support). | Restorative validity agenda, where validity is reframed as whether evaluation contributes to the strength/health/worth of those involved (evaluator, evaluated, and process), rather than only technical adequacy. | Transformative participatory evaluations | Participatory and justice-oriented evaluation approaches, such as co-learning sessions; community consultations; use of oral tradition as ‘sacred data’; iterative questioning adapted from restorative justice practice to interrogate power, participation, and repair. |
| Erb et al. (2025) [5]Relational systems and the principles of self-determination, reciprocity, and mutual benefit: enacting evaluation. (Peer-reviewed paper) | Focuses on the Network Environment for Indigenous Health Research evaluation approach (Environmental Evaluation Framework and annual reports), situated in British Columbia | The paper examines how evaluation can be enacted within a networked Indigenous health research environment by attending to relational systems. It interrogates an existing evaluation framework and associated reporting to identify where evaluation practice aligns (or fails to align) with Indigenous principles such as self-determination, reciprocity, and mutual benefit. | Relational systems framing; principles of self-determination, reciprocity, mutual benefit; BC NEIHR Environmental Evaluation Framework as the object of critique. | Indigenous self-determination and relational accountability. | Critical content analysis of evaluation texts (framework + annual reports) supported by a literature review. |
| Van der Puije (2205)[6]. Decolonising Evaluation Practice in International Development Cooperation Through an African Religion Lens (Peer-reviewed paper) | Decolonial critique of international development evaluation through African religion as an epistemic/ontological frame; draws on cases from Ghana, South Africa, and Botswana. | The paper argues that mainstream development evaluation frequently marginalises African religious worldviews, producing epistemic exclusion when ‘evidence’ is restricted to secular, technocratic forms. It proposes an ‘African religion lens’ as a decolonising corrective, treating spirituality and communal moral order as constitutive of social life and thus of programme meaning and value. | Decolonial evaluation critique through African religion lens (as evaluative frame); | Decolonial theory and African relational ontology. | Reframing evaluation criteria and redefining admissible ‘evidence’ and authority. |
| Puije and Satzinger (2025) [7]Decolonising international development evaluation: rethinking OECD DAC evaluation criteria from a decolonial perspective. (Peer-reviewed paper) | International development | The paper argues that widely institutionalised evaluation criteria in development reflect a history of donor governance that can reproduce colonial power relations through what is measured, valued, and made legible. It proposed ‘rethinking’ is oriented toward epistemic justice, and evaluation criteria should be re-specified in ways that make room for locally grounded ontologies, relational accountability, and historically situated understandings of value and harm. | Decolonial reframing of OECD Development Assistance Committee criteria; evaluation criteria as a site of power | Decolonial theory, epistemic injustice. critique of donor governance and audit logics embedded in ‘standards’. | NA |
| Sehgal et al. (2024). [8]Advancing health equity for Indigenous people in Canada: development of a culturally safe patient complexity assessment framework for primary care  (Peer-reviewed paper) | Health equity framework development in Canada | Develops Indigenous-centred complexity framework incorporating culture, resilience and structural inequities. The final framework comprises 27 concepts across nine domains and deliberately adds domains/concepts often erased in standard tools, including adverse life experiences, resilience and culture, and healthcare violence. | Culturally safe patient complexity assessment framework and Indigenous-centred concept development. | Health equity and cultural safety frameworks, attention to structural violence and racism in healthcare, and epistemic authority of patient/community knowledge in defining constructs. | Participatory and consensus-based framework development. |
| Stefanski et al. (2025). [9]Indigenous Evaluation: A Scoping Review of Indigenous Evaluation Methods and Guiding Principles  (Peer-reviewed paper) | Indigenous evaluation spanning multiple settler-colonial contexts | The paper synthesises what Indigenous evaluation ‘requires’ by cataloguing principles and methodological approaches. It identified 13 guiding principles (e.g., collaboration, respect, relationships, self-determination, reciprocity, sovereignty, responsibility/accountability, relevance). | Indigenous evaluation guiding principles | Indigenous ways of knowing and culturally grounded paradigms, sovereignty and self-determination as evaluative foundations. | The paper identified 15 Indigenous evaluation methodologies (e.g., participatory/community-based, storytelling, decolonising, self-location/reflexivity, talking circles, ethical space, two-eyed seeing, trauma-informed approaches). |
| Office of Planning, Research, and Evaluation (OPRE) (2024)[10]. Engaging Indigenous Populations in Evaluation Through Indigenous Methodologies. (Grey Literature) | Guidance for evaluation in Tribal contexts in the context of United States. | It provides operational guidance for Indigenous methodologies across planning, implementation, analysis and dissemination. | Indigenous evaluation frameworks and participatory evaluation. | Indigenous epistemologies and sovereignty-oriented ethics | Participatory, community-engaged evaluation through staged recommendations across evaluation phases, emphasis on governance, relationship-building, community-led interpretation, and dissemination for community benefit. |
| Dutta et al (2024) [11]Decolonizing Impact Through the Culture-Centered Approach to Health Communication: Mobilizing Communities to Transform the Structural Determinants of Health.  (Peer-reviewed paper) | Health communication and impact evaluations in contexts of racial colonialism, settler colonialism, and neoliberal capitalism, particularly the indigenous land occupation in Aotearoa/New Zealand, migrant workers in Singapore, African American communities. | The paper critiques the dominant evaluation ideology (rooted in Cold War ideology/neoliberalism) for narrowly measuring individual behaviour while obfuscating the underlying colonial and capitalist structures that cause health disparities. The Culture-Centered Approach (CCA) reframes impact as decolonisation- an explicit anti-colonial, anti-capitalist endeavour- where success is evidenced in the transformation and dismantling of oppressive structural barriers | Culture-Centered Approach (CCA) Theory-Method-Praxis Model: A meta-theoretical framework where health disparities result from structural oppression. CCA’s purpose is to build voice infrastructures to achieve social change and structural transformation | Critical Theory, Postcolonialism, and Subaltern Theory. Grounded in a decolonising reading of health that critiques Whiteness as a structure that upholds the colonial-capitalist formation. The axiological goal is social justice. | Anti-colonial/Anti-capitalist Activism and Culturally Centered Organizing. Analysis uses a multi-level socio-ecological model (micro, meso, macro) to interrogate how structures operationalize marginalization.  Methods involve co-creating voice infrastructures (e.g., community advisory boards, co-constructive ethnography, photovoice, and poetry/narratives). Also emphasises "body on the line" (the researcher's body as a site of active activist solidarity and resistance) |
| Melro et al (2023) [12]A scoping review of frameworks utilized in the design and evaluation of courses in health professional programs to address the role of historical and ongoing colonialism in the health outcomes of Indigenous Peoples.  (Peer-reviewed paper) | Health professional education in settler colonial contexts (Canada, Australia, New Zealand, United States) | The paper discusses frameworks and evaluation of educational interventions addressing colonialism in health education. It reviewed 14 articles focused on outcomes like beliefs, attitudes, and emotional reactions related to historical and ongoing colonialism. Educational content addressing colonialism can cause resistance among students. | The Four R framework (Respect, Reciprocity, Relevance, and Responsibility) to ethical and culturally appropriate engagement with Indigenous Peoples | Informed by Indigenous worldviews and an understanding of colonial history. | Evaluated changes using validated self-report measures and qualitative methods; qualitative methods focused on identifying changes in learners' causal beliefs about the role of colonialism. |
| Povey et al (2023) [13]An emerging framework for digital mental health design with Indigenous young people: a scoping review of the involvement of Indigenous young people in the design and evaluation of digital mental health interventions.  (Peer-reviewed paper) | Digital mental health (dMH( in  Australia, New Zealand, Canada, USA; Digital Mental Health for Indigenous youth | The paper discusses the design and evaluation of dMH interventions involving Indigenous young people, by developing an integrated framework by aligning participatory design principles with the Aboriginal and Torres Strait Islander Quality Appraisal Tool (QAT). | Emergent Framework for dMH development/evaluation: Structured around four domains: governance, engagement, partnerships, and research translation. | Guided by Indigenous research principles; Indigenous governance, cultural respect and adaptation, capacity building, and explicit measures to ensure community benefit and intellectual property protection. In addition, Povey et a highlight  participatory design, and recognition of shared colonial histories. | Use of the Aboriginal and Torres Strait Islander QAT to assess best practice. Consultation with Senior Indigenous Research Officers to ensure analysis is informed by Indigenous worldviews. |
| Sutthibut et al (2021) [14]Development of an Assessment Tool of Menstrual-Cycle-Related Signs and Symptoms Based on Thai Traditional Medicine Principles for Evaluation of Women's Health.  (Peer-reviewed paper) | Menstrual health and traditional medicine among Thai women/Thai Traditional Medicine (TTM) | The paper describes developing and validating the first questionnaire (MCSQ) based on TTM principles to assess MCS severity and identify problematic internal organs/systems. Showed acceptable validity and reliability using rigorous methods (Delphi, Exploratory Factor Analysis (EFA)). | Thai Traditional Medicine (TTM) principles: TTM theoretical framework regarding pathogenesis (four elements: Earth, Water, Wind, Fire) used for constructing and validation. | TTM theoretical conceptual construct. | Delphi method used to reach consensus among TTM experts on essential content. Quantitative validation (CVI, EFA, PCA). |
| Bailie et al (2020) [15]Using developmental evaluation to enhance continuous reflection, learning and adaptation of an innovation platform in Australian Indigenous primary healthcare.  (Peer-reviewed paper) | Developmental evaluation (DE) in Primary Health Care (PHC)in within the settler colonial context of Australian Indigenous communities. | The paper documents how DE was systematically applied to enhance the functioning of the Centre for Research Excellence in Integrated Quality Improvement in Indigenous Primary Health Care (CRE-IQI). The approach aligned strongly with DE principles, focusing on improving rather than proving. The real-time feedback led to concrete adaptations, such as restructuring the leadership to ensure Indigenous control over future research networks | Developmental Evaluation as an approach suited for innovative or emergent environments that applies complexity concepts and systems thinking to enhance innovation and use. Its focus is on developmental purpose to support learning and adaptation | This paper drawa on Systems Thinking and Complexity Concepts (attending to non-linearity, feedback, and emergence). It is grounded in the need to address health inequities resulting from the pervasive legacy of colonisation, land dispossession, and ongoing racial discrimination. | Co-creation (developed and refined drawing on multi-stakeholder input). Use of continuous learning cycles analogous to Continuous Quality Improvement (CQI) methods (Plan- Do- Study- Act). Intentional valuing of Indigenous knowledge by centering participant voices |
| Lawrence and James (2019) [16]Good Health and Wellness: Measuring Impact Through an Indigenous Lens.  (Peer-reviewed paper) | Good health and wellness  within the settler colonial context of United States  Indian Country (Tribes and Tribal Health Organizations) | The paper discusses measuring impact through an Indigenous Lens (GHWIC). The evaluation tracks performance measures showing improved access to healthier foods and physical activity. Approach prioritizes recipient feedback and approval before dissemination. | GHWIC Evaluation Approach / Indigenous Lens: Evaluation must be meaningful to Tribal audiences and federal funders. | Personal and Tribal sovereignty. | Program evaluation approach reflecting a 3-tiered structure (local, regional, national). Iterative communication; obtaining approval from recipients regarding products before dissemination; soliciting recipient feedback on evaluation reports. |
| Cargo et al (2019) [17]Strategies to support culturally safe health and wellbeing evaluations in Indigenous settings in Australia and New Zealand: A concept mapping study.  (Peer-reviewed paper) | Indigenous health and wellbeing evaluations in the settler colonial contexts of Australia and New Zealand. | The study empirically identified practical strategies for ensuring culturally safe evaluations in Australia and New Zealand. It found that while strengthening evaluator characteristics (e.g., self-awareness, integrity) is generally viewed as highly important and achievable, changing systemic factors—such as ensuring funding is responsive to community needs and priorities and addressing the conduct of commissioning—were consistently rated as the least achievable strategies in both countries. The findings underscore the centrality of relational accountability | Culturally Safe Evaluation (Kawa whakaruruhau): Defined as evaluation practices that meet the needs of Indigenous people from an Indigenous cultural worldview. It requires evaluators to be critically self-aware of their cultural biases and power and to privilege equity in power relationships | The evaluation is grounded in an Indigenous epistemology of relational accountability (connecting people, environment, and the spirit world) and often draws specifically on Kaupapa Māori theory in the New Zealand context. It addresses the need to confront the pervasive legacy of colonisation and subsequent racial discrimination causing health inequities | Participatory processes and Indigenous leadership are advocated to ensure self-determination and community ownership. The process requires critical self-reflection/reflexivity (especially for non-Indigenous evaluators) to identify personal biases and white privilege. Evaluation must prioritize community benefit and secure community endorsement for publication and reports (downward accountability) |
| Rarere et al (2019) [18]Critical reflection for researcher-community partnership effectiveness: The He Pikinga Waiora process evaluation tool guiding the implementation of chronic condition interventions in indigenous communities.  (Peer-reviewed paper) | Non-communicable diseases in indigenous communities (Māori) of New Zealand. | The study assess how well two research-community partnerships adhered to the He Pikinga Waiora (HPW) Implementation Framework—a tool centered on Indigenous self-determination and critical reflection—while co-designing chronic condition interventions for Māori communities. Alignment to the HPW framework was strong, especially regarding community engagement. | The paper uses He Pikinga Waiora (HPW) Process Evaluation Tool/Framework to evaluates systems thinking, community engagement, cultural centredness, and reflexivity. | The need to eliminate health inequities for Māori. | Use of a measurable tool to assess reflexivity (power/privilege issues) and community participation. Community emphasised that the "how" (respectful engagement) was more important than the "what". |
| LaFrance and Nicols (2008) [19]Reframing evaluation: Defining an indigenous evaluation framework  (Peer-reviewed paper) | Evaluation methods in American Indian Higher Education Consortium (AIHEC)/Tribal Colleges | The paper defines core values for an Indigenous Evaluation Framework (IEF). It describes the process of gathering Indigenous wisdom and perspectives on evaluation using tribal protocols and non-traditional discussion formats. | Indigenous Framework for Evaluation (IEF): Must capture the holistic concept of "becoming... living within this larger frame and in harmony, and in peace and in that sense of place". | Traditional Native cultural epistemologies; Indigenous perspective. | Use of cultural protocols (smudging, tobacco offering, food gifts). Conversation flowed naturally (not Q&A format); focus on sharing traditional values and stories. |
| Whitau and Ockerby (2019) [20]Yarning with the Stars Project: An Indigenous evaluation protocol for a sport for development and peace program  (Peer-reviewed paper) | Sports for Development among Aboriginal and Torres Strait Islander girls. | This paper outlines the Indigenous evaluation protocol for the "Shooting Stars" program, which uses netball to promote the education, health, and well-being of Aboriginal and Torres Strait Islander girls. The evaluation focuses primarily on the Yarning with the Stars project, which uses yarning circles as the primary data collection method, developed collaboratively over 18 months. The process provides a platform for student voice and self-determination and aims to enable communities and participants to drive the direction and content of their local program | Yarning with the Stars Project Evaluation Protocol: A model of Indigenous evaluation methods that utilizes yarning circles to gain qualitative evaluation data. It is designed as a culturally responsive, decolonized methodology that emphasizes relationship building and epistemological equality between Western, Aboriginal, and other ways of knowing | Relatedness Theory. This framework is situated within Aboriginal ways of knowing, being, and doing, emphasizing inclusivity and subjectivity. It also incorporates core Indigenous research principles such as respect, inclusive decision making, equality of input and control, and privileging Indigenous voices, perspectives, and knowledge systems | Yarning Circles (used as the primary mode for data collection to provide a culturally responsive research space and build relationships). Culturally responsive, decolonized methodology. Focuses on self-determination and ensuring communities and participants drive the direction and content of their program.  The methods blend Indigenous practices (yarning circles, storytelling, knowledge-sharing through art and objects) and non-Indigenous techniques (scaffolding learning, thematic analysis, audio-recording, and transcription). |
| Chilisa et al (2016) [21]Decolonizing and Indigenizing Evaluation Practice in Africa: Toward African Relational Evaluation Approaches  ((Peer-reviewed paper) | Evaluation methods,  core theoretical & foundational paradigms in Africa (Bantu communities) | The paper discusses the African relational evaluation approaches, particularly the Ubuntu-inspired approaches. It advocates moving evaluation focus from external goals to the valued needs and priorities of the local people (e.g., poverty alleviation, employment). Illustrates the "valuing branch" of evaluation | African Relational Evaluation Approaches / Ubuntu: Utilises elements of collaboration, togetherness, and consensus building. Evaluator must undergo transformation to become a Muntu (person of the community) to access deep knowledge | Ubuntu philosophy (I/We relationship). This core principle dictates that an individual's identity is defined by their place within the community, which includes relationships not only with other people but also with the living and the non-living, thereby promoting values of collective responsibility, cooperation, love, and harmony | Use collaborative planning and execution; gaining rapport by sharing family, history, clan, and totem(a traditional symbol used to represent and describe lineage, ancestry, and identity within certain African and Indigenous communities); evaluator transformation |
| Chilisa and Mertens (2021) [22]Indigenous Made in Africa Evaluation Frameworks: Addressing Epistemic Violence and Contributing to Social Transformation  (Peer-reviewed paper) | Conceptual/Theoretical evaluation discussion in Africa/Global South (LMIC) | The paper discusses the Indigenous Made in Africa evaluation framework, addressing epistemic violence and self-determination. It examines how the reliance on dominant Global North paradigms constitutes epistemic violence and fails to address local contexts and priority needs. | Indigenous Made in Africa Evaluation (MAE): A relational evaluation approach aimed at restoration, protection, and revitalization of community knowledge. | Ubuntu ("I am because we are"), along with the postcolonial and transformative frameworks. | Reflexivity (continuous reflection on position within power structures). Evaluation needs to be responsive to prioritized needs identified by communities. |
| Chilisa et al. (2017) [23]Community Engagement with a Postcolonial, African-Based Relational Paradigm  (Peer-reviewed paper) | Indigenous scholars and communities marginalized by Euro-Western paradigms. It proposes a universal Afrikology epistemology that goes beyond geographical borders | The article argues for a conceptual shift to advance Indigenous paradigms to achieve epistemological equality for marginalized people. The process is driven by explicit decolonisation intent and aims to revitalize Indigenous knowledge and restore self-identity. It advocates for multi-epistemological research partnerships to dismantle deficit theorising. It defines the African-based relational paradigm and postcolonial methodology for decolonising research and promoting equal status for indigenous knowledge systems. | Postcolonial Indigenous Methodology Framework / African-based Relational Paradigm: A framework rooted in African philosophical assumptions of connectedness and relatedness (ontology of "we-ness" and "us-ness"). The framework shields the protection, restoration, and revitalization of local knowledge | It is framed within a postcolonial Indigenous paradigm and an African-based relational ontology and epistemology. Grounded in the intent of decolonisation, its epistemology, Afrikology promotes harmony and balance and a fearless aspiration for new paradigms | Relational Indigenous Methodologies. Focus on Community Engagement. Use of Integrative Approach and Third Space Methodologies to balance borrowing Western and Indigenous methods. Requires non-Indigenous researchers to engage in self-reflection and explicitly address power differentials between knowledge systems. |
| Dlakavu et al (2022) [24]Decolonising and indigenising evaluation practice in Africa: Roadmap for mainstreaming the Made in Africa Evaluation approach  (Peer-reviewed paper) | Conceptual/Theoretical roadmap in Africa/Global South Evaluation | The paper advocates for the mainstreaming the Made in Africa Evaluation (MAE) approach. MAE is positioned as the continent’s decolonisation and indigenisation project, seeking Afrocentric evaluation practice based on African developmental values. | Made in Africa Evaluation (MAE) approach: A paradigm that is contextually relevant, relational, and emanates from the realities and values of Africans. | The theoretical underpinning is grounded in Afrocentric values and approaches to decolonisation and indigenisation. | N/A (Focus on institutional policy roadmap). |
| Kelly and Htwe (2023) [25]Decolonizing Community Development Evaluation in Rakhine State, Myanmar.  (Peer-reviewed paper) | Community Development Program aimed at reducing poverty and strengthening peace between ethnic Buddhist Rakhine and Muslim Rohingya communities. | The paper documents the highly complex journey toward decolonizing evaluation, asserting that simple "capacity building locals around Western ways" or translating findings is reductionist and insufficient. The core challenge is navigating vast and layered power imbalances and shifting evaluation from upward donor compliance to locally led learning. The evaluator's role shifted from external expert to "critical friend" to intentionally reduce the influence of Western ways | Decolonizing based on a process focused on redistributing power and transforming the evaluator's mindset. It draws aspirational alignment from frameworks like “Empowerment Evaluation” and “Indigenous Evaluation”, which emphasise local design, data gathering, analysis, and interpretation. | Decolonization Theory (citing Ngũgı̃ wa Thiong’o’s call to decolonize the mind and Tuck & Yang's "Decolonisation is not a metaphor"). Focus on critical consciousness-raising and challenging the supremacy of Western ontologies and epistemologies | Critical Reflection/Collaborative Auto-ethnography. Advocacy for the use of local knowledge and interpersonal skills over formal Western qualifications. Intentional focus on power dynamics and reflexivity regarding the evaluator's own biases, particularly concerning the internal oppression dynamics (Rakhine/Rohingya). |
| Brown and Di Lallo (2020) [26]Talking Circles: A Culturally Responsive Evaluation Practice.  (Peer-reviewed paper) | a case study of the Stollery Awasisak  the Stollery Awasisak (Cree word for children) Indigenous Health Program in Canada. | The article proposes the Circle, an ancient method adapted from Restorative Justice (RJ) contexts, as a values-based and relational process that creates safe spaces to nurture relationships and establish norms. It is designed to empower participants and achieve more just evaluations that "do good" | The Circle Method / Talking Circles: A structured but flexible method rooted in traditional ways, intentionally designed not to privilege one worldview over another, aligning with Culturally Responsive Evaluation. The evaluator takes on the role of the Evaluator-Circle Keeper (E-CK) | Culturally Responsive Evaluation (CRE). It is grounded in theories such as Critical Race Theory (CRT) and Transformative theory. The axiological goal is to ensure work supports social justice and leads to personal and professional transformation for the evaluator | Culturally Responsive Evaluation (CRE), Restorative Justice (RJ) Principles. Requires the evaluator to engage in critical reflection on their own biases and practice  The core method is the Talking Circle (or "Circle"), a dialogic process utilised as an alternative to traditional focus groups. |
| Pant et al. (2022) [27]Decolonising Global Health Evaluation: Synthesis from a Scoping Review.  (Peer-reviewed paper) | Global Health Evaluations | The scoping review provides a synthesis of factors and themes associated with decolonising global health evaluation. The review concludes that decolonisation is a transformative, liberation movement aimed at restructuring power imbalances and establishing equitable, mutually beneficial, and reciprocal partnerships. | Decolonisation of Global Health Evaluation: Factors categorised across Micro, Meso, Exo, and Macro socio-ecological levels (e.g., individual mindsets to collective culture). | Informed by transformative and liberation paradigms, emphasising the repair and restoration of trust against a historical backdrop of mistrust | The approaches include prioritising recipients to lead effort and intentionality in curricula, methods, and theoretical frameworks. It also promotes reflexivity concerning power, privilege, and gaze. |
| Renmans et al. (2022) [28]Realist Evaluation in Times of Decolonising Global Health  (Peer-reviewed paper) | Focuses on Realist Evaluation (RE) in global health, particularly in low- and middle-income countries (LMICs). It specifically addresses evaluations conducted by "external researchers" (whether foreigner or not, who are not from the communities under consideration | Discussing the potential of Realist Evaluation (RE) to contribute to the decolonisation of global health by involving local stakeholders and putting their assumptions on an equal footing with substantive social science theories. However, this potential is often thwarted in practice by external researchers who display a lack of acknowledgment of power imbalances, fail to recognize the existence of different but equivalent ontologies, and over-rely on Western-based theories and concepts. The persistence of strict hierarchies of knowledge in contemporary science is a major obstacle | Realist Evaluation (RE), a theory-driven approach (inspired by scientific realism), seeks to identify generative mechanisms and underlying (social) structures. The paper suggests RE be combined with emancipatory and participatory practices to actively contribute to decolonization | The paper is driven by the decolonization movement in global health, which critiques the universalisation of Eurocentric ways of being and doing. It warns against the use of research concepts based on WEIRD societies (Western, Educated, Industrialised, Rich, and Democratic societies). The movement focuses on shifting the locus of power towards local stakeholders | Requires active and critical engagement with power inequities. External researchers must recognise that their knowledge production is constrained by their social, economic, and cultural position. Failure to acknowledge power differentials means RE risks perpetuating inequalities. It is argued that RE methods should prioritise participatory (action) research methods (currently underused in RE) to ensure rival theories and feared unintentional outcomes receive equal attention (adversarial stakeholder participation) |
| Igwe et al. (2022) [29]Decolonising Research Approaches towards Non-Extractive Research.  (Peer-reviewed paper) | Global South/Indigenous communities | The paper discusses non-extractive research approaches and ethics. Research methodology must allow a mutually constitutive relationship between method and content. Non-extractive research focuses on participatory principles and research ethics. | Culturally Inclusive Research Approaches Model, which is built on the six Rs: respect, relevance, reciprocity, responsibility, relationships, and relationality. | The theoretical underpinning of this paper is situated within a postcolonial paradigm. | The approach adopted in this paper requires a focus on collaborative autoethnography and self-reflection. |
| Zwiener-Collins et al. (2023) [30]Decolonising Quantitative Research Methods Pedagogy: Teaching Contemporary Politics to Challenge Hierarchies from Data.  (Peer-reviewed paper) | Research  Higher Education (undergraduate politics curricula) in the UK and globally, driven by movements like Black Lives Matter and the ongoing project to decolonise the curriculum | Decolonising the teaching of quantitative research methods by challenging the perception of quantitative data as value-free and neutral, and exposing the authoritarian, hierarchical, and hegemonic nature of data production. Quantitative methods and data reproduce existing power relations, Concepts considered universal in QM (like 'democratic support' or 'GDP') must be critically deconstructed in the classroom to reveal their Eurocentric biases and False Universalism. The goal is to produce critical data users who are aware of the origins and consequences of data, rather than just non-critical users intimidated by numbers | QuantCrit (Quantitative Critical Race Theory): A framework derived from Critical Race Theory (CRT) that challenges the idea of statistics as neutral, acknowledging that data sources carry hegemonic baggage and that statistical analysis can be used to advance racial equity | Rooted in decolonisation theory (which seeks to overhaul the very idea of higher education and dismantle Eurocentric knowledge production), and Critical Pedagogy. It resists the positivist paradigm where QM is perceived as value-free and producing generalizable knowledge | Anti-Hegemonic Practice. Strategies include actively resisting Eurocentrism (e.g., by including Global South concepts and data), acknowledging positionality, and challenging the use of universal concepts by questioning their cross-cultural equivalence and non-universal validity, which is particularly evident in comparative political sociology  Advocates for a decolonial approach that integrates four activities: resisting Eurocentrism, showing systematic Eurocentrism, integrating race critical thinking, and encouraging critical data use/creation |
| Dighe et al (2023) [31]Deconstructing the Imperial Episteme: Decolonizing Knowledge Production in Program Evaluation  (Peer-reviewed paper) | International development, focusing on evaluation in Global South and with Indigenous communities in the Global North. The intent is to contribute to theory-building by addressing the colonial foundations of Northern social science thought | Advancing epistemic decolonization in program evaluation by deconstructing the "imperial episteme" (colonial influences on knowledge construction) rooted in Western social theory. Decolonisation is viewed as a paradigmatic shift that questions power hierarchies embedded in Western social theory. The paper identifies four apparatuses of colonization of knowledge (Historical Eurocentrism, Analytical Bifurcation, Historicism, and False Universalism) that must be dismantled to make space for knowledge from the Global South. The goal is to move beyond merely adapting Western practices, insisting that new evaluation frameworks must be rooted in Global South experiences | Imperial Episteme (Colonial Ways of Knowing): A concept from postcolonial theory that describes how Western social science thought, developed in colonizing nations, systematically promotes specific ontological, epistemological, and axiological assumptions that marginalize the experiences and knowledge of the Global South and Indigenous peoples | Postcolonial Theory (provides an "outsider" perspective to critique Western social science). It is driven by the realization that evaluation theory is incomplete because it has not recognized or validated the experiences of colonialism as a distinct social phenomenon. Concepts like Indigenous Evaluation Framework (IEF) and Made in Africa Evaluation (MAE) are seen as concrete examples of building new frameworks outside the colonial frame | Two-pronged approach for decolonization: 1) Material Realm (creating new frameworks based on Global South experiences); 2) Epistemic Realm (deconstructing Eurocentric knowledge). Strategies include Geolocating Evaluation Knowledge (naming the origin and philosophical stance of knowledge to challenge its presumed universality) and Troubling the Categorical Schema (questioning the meaning and relevance of universal terms like "civil society")  The approach advocates for recognizing non-Western ontological and epistemological frames as equal, rather than "alternative," to Western frameworks. It uses theoretical tools from outside the traditionally-defined boundaries of evaluation |
| Goldman et al (2024) [32]Decolonising national evaluation systems.  (Peer-reviewed paper) | African National Evaluation Systems (NESs) (Benin, South Africa) | Rethinking National Evaluation Systems (NESs) for transformation by analysing how African NESs are addressing decolonisation, noting that many still perpetuate neo-liberal models and rely heavily on lengthy written reports. | Decolonised NES (Implicit framework based on African approaches). | Need for transformation toward "fifth paradigm", new evaluation paradigm emerging in evaluation theory and practice, one that builds on and moves beyond existing paradigms by centring decolonial, social-ecological, and system-based thinking. It prioritises community participation, challenges entrenched power dynamics, and integrates learning and equity to address social and environmental challenges. | Investing considerably in relationships with government stakeholders. |
| Goforth et al. (2022) [33]Incorporating the Indigenous Evaluation Framework for Culturally Responsive Community Engagement  (Peer-reviewed paper) | Native American students in school-based mental health | The paper discusses using the Indigenous Evaluation Framework (IEF) and Tribal Critical Race Theory (TCT) to guide research. It argues for the use of collaborative autoethnography to establish trustworthiness and identify themes related to IEF core values, particularly the centrality of relationships. | Indigenous Evaluation Framework (IEF) is a culturally responsive model that integrates Indigenous epistemologies and Tribal Critical Race Theory. It centres community context and relationships, values immersion and presence, and promotes authentic partnership through shared power. The framework also emphasises storytelling, respect for Tribal sovereignty, and researcher reflexivity in acknowledging positionality and history. | Tribal Critical Race Theory (TCT), examining the intersections of race, colonisation, and Indigenous sovereignty to highlight systemic inequities affecting Native communities. | Approaches combine collaborative autoethnography and self-reflexivity, using individual reflection and group discussion as data, with an emphasis on centring relationships and accountability. |
| Kane and Thomas (2023) [34]Ubuntu and Afrofeminism for Decolonizing Evaluation.  (Peer-reviewed paper) | African contexts, specifically aiming to inform the discourse and practice of Made in Africa Evaluation (MAE). It addresses the masculinist, imperialist, and modernist ideals inscribed in institutions like government rationality | The framework seeks to dismantle the imperial episteme and hierarchical logics that persist in evaluation. It argues that combining Ubuntu, which champions humanness and interdependence, with Afrofeminist critique allows evaluators to foreground intersectionality and problematize categorial, dichotomous, and hierarchical logics, including methodological hegemonies. This approach resists "neoliberal Band-Aid approaches" and aims for liberatory praxis | Ubuntu and Afrofeminism: A framework asserting that evaluation must be rooted in the African relational paradigm (Ubuntu) but filtered through a decolonizing Afrofeminist lens to expose and dismantle systems of oppression that persist post-colonially, particularly around gender and class | The work is rooted in the African-based Relational Paradigm and Ubuntu philosophy ("I am because we are"). It draws on Postcolonial Theory to resist the "colonisation of the mind" (epistemic coloniality). The approach is driven by a decolonizing intent and the axiological goal of social justice and emancipation | Made in Africa Evaluation (MAE) and Culturally Responsive Evaluation (CRE) were highlighted. The paper advocates for evaluation praxis rooted in relationality and communitarian framings, prioritizing methods that reflect the "moral economy". Methods must allow for consensus building and leverage a plurality of evidence. |
| Bowman-Farrell (2018) [35]Looking backward but moving forward: honouring the sacred and asserting the sovereign in Indigenous evaluation  (Peer-reviewed paper) | US Indigenous/Tribal Nations | Honoring the sacred and asserting the sovereign in Indigenous evaluation  Calls upon the evaluation profession to actively dedicate resources to reverse the historic and current impact of structural racism and colonialism. | Culturally Responsive Indigenous Evaluation (CRIE) (Implicit). | Tribal sovereignty; self-determination; decolonizing the mind. | Focus on building evaluator capacity within Indigenous contexts. Decolonized methods replace colonial methods with original Indigenous teachings. |
| Millet (2019) [36]Ukombozi means liberation: A case for decolonizing global health research, methodology, and praxis  (Peer-reviewed paper) | Dar es Salaam, Tanzania, focusing on the cultural and linguistic adaptation of the Ukombozi program, which addresses substance abuse, HIV/AIDS, and gender-based violence. | The paper discusses the use of Community-Based Participatory Research (CBPR) for cultural and linguistic adaptation of a program. The research contributes practical demonstrations of how to decolonize global health work by undoing the dominance of Western epistemologies. Key learnings included navigating institutional gatekeeping, indigenizing data definition, and removing sanctions from the research process. The ultimate goal is to reclaim, repair, and restore power and sovereignty in health governance to previously colonized populations. The program name itself, Ukombozi, means liberation | Alter/Native Methods: A term used for paths of inquiry that subvert colonial constructs in global health work to serve decolonizing purposes. These methods align work with values of equity and sovereignty in health governance | Rooted in a postmodern perspective where research is a representation of reality. The work draws on postcolonial theory, critiquing the "Imperial gaze". It is driven by the principle of liberation (Ukombozi) and resistance against neocolonial structures | Community-Based Participatory Research (CBPR), which emphasizes First Voice (the researcher does not speak for others), building relationships, and shifting the researcher's role to an active agent of resistance. The methodology employed "Alter/Native" methods to decolonize the research process. Specific methods included a critical ethnography of the program adaptation process, indigenizing data collection, and using Ngonjera as Methodology (a dialogue form) to present themes and provoke the audience. The approach focused on shifting researcher positionality by adopting Trickster as a thinking companion and engaging in active "Grounding In" |
| Kerr (2012) [37]Kaupapa Maori theory-based evaluation  (Peer-reviewed paper) | Aotearoa/New Zealand, focusing on evaluations of programs and services for the Māori (Indigenous peoples of New Zealand). The context addresses the historical colonization and the need to address wide-ranging disparities experienced by Māori | The paper presents Kaupapa Māori Evaluation (KME) as a counter-hegemonic practice that developed as an assertion of Māori sovereign status and a means to reclaim a future founded within a Māori worldview. It uses theory to define the purpose and parameters of evaluation, emphasizing that the philosophical foundations of KME are crucial for understanding the complexities of embracing multiple socially constructed realities. KME is guided by six core principles and is ultimately aimed at achieving positive transformation for Māori | Kaupapa Māori Theory-Based Evaluation (KME): A dynamic and coherent theory of praxis where thinking and practice work together. It is guided by six principles: Control (self-determination), Challenge (analysis of power), Culture (Māori as normative), Connection (relationship-based knowledge sharing via whānau), Change (transformative outcomes), and Credibility (highest quality standards for Māori) | Rooted in Kaupapa Māori Theory which stems from Māori worldview and connects to critical social theory and a constructivist epistemology. Its foundational argument is based on Māori rights to self-determination (Tino Rangatiratanga) as guaranteed by the Treaty of Waitangi. It shares strong alignment with Transformative Evaluation approaches, which seek to use evaluation to provide solutions to social problems | Collaborative, Transformative, and Context-Specific Evaluation requires strict adherence to ethical codes of conduct such as Kia tūpato (be cautious/culturally safe) and Manaaki ki te tangata (share and host people, be generous). Emphasis is placed on reciprocity (koha) to ensure all participants receive something of value in return. |
| Cram (2016) [38]Lessons on decolonising evaluation from Kaupapa Māori Evaluation  (Peer-reviewed paper) | Conceptual/case analysis in Kaupapa Māori (Aotearoa/New Zealand) | The paper discusses decolonising evaluation using lessons from Kaupapa Māori Evaluation (KME) based on collective interdependence (whakapapa) and Indigenous knowledge. Requires evaluators to act with Kia tūpato (be cautious/careful). | Kaupapa Māori Evaluation (KME) utilizes "community-up" practices and the principles of Control, Challenge, Culture, Connection, Change, and Credibility. | Kaupapa Māori theory; resistance to capitalist "economy of exploitation". | Seven community-up research practices.  1. Aroha ki te tangata (a respect for people).  2. Kanohi kitea (the seen face; that is, present yourself to people face to face).  3. Titiro, whakarongo … korero (look, listen … speak).  4. Manaaki ki te tangata (share and host people, be generous).  5. Kia tupato (be cautious or be careful).  6. Kaua e takahia te mana o te tangata (do not trample over the mana of the people).  7. Kaua e mahaki (do not flaunt your knowledge or Be humble). |
| Boadu and Ile (2024) [39]Beyond the Buzzword: A Framework for an Indigenous Relational Evaluation in Traditional Communities in Ghana.  (Peer-reviewed paper) | Traditional communities and local government areas in Ghana, Africa. The study addresses the lack of indigenously driven evaluation practices in Ghana | The paper argues for developing a clear, concise, and practical framework for Indigenously Responsive Evaluation (IRE) methods and philosophies rooted in Ghanaian cultural realities. Indigenous knowledge and cultural ethos are effective in community-based development evaluation, operating through communal and relational patterns. IRE centers principles like Ubuntu ("I am because we are"), consensus building, and self-organization. The methodology aims to supplement mainstream evaluation by being culturally situated and recognizing that evaluation is a collective activity rooted in cultural tenets | Indigenously Responsive Evaluation (IRE) Framework / Synthesized Indigenous Participatory M&E Framework: A practical approach guided by African philosophical assumptions, prioritizing Indigenous Knowledge Systems (IKS) and addressing the lack of indigenously driven concepts in contemporary evaluation.  The goal is to develop an Indigenously Responsive Evaluation (IRE) framework rooted in consensus building and communal values. Indigenous evaluators prioritize the "why" and "how" of evaluation over the "who" and "what" common in Western models. | Rooted in the African-based Relational Paradigm and Ubuntu philosophy ("I am because we are"). The approach aligns with the Made in Africa Evaluation (MAE) mantra and incorporates principles from Culturally Responsive Indigenous Evaluation (CRIE) | Culturally Responsive Indigenous Evaluation (CRIE). Participatory Evaluation (PM&E). Emphasizes Consensus Building and leveraging Indigenous knowledge systems (IKS) and cultural ethos. Requires the evaluation to be anchored on principles of trust, transparency, and accountability. |
| Boadu and Ile (2023) [40]Evaluation innovation in Africa: Towards indigenously responsive evaluation (IRE) philosophies, methods and practices in Ghana  (Peer-reviewed paper) | Traditional communities and local government areas in Ghana, Africa. The study addresses the "evaluation development impasse" and the lack of indigenously driven evaluation practices in Ghana. | The paper discusses developing a clear, concise, and practical framework for Indigenously Responsive Evaluation (IRE) methods and philosophies rooted in Ghanaian cultural realities, particularly for community development programs  The study finds that Indigenous knowledge and cultural ethos are distinct and effective in community-based development evaluation, operating through communal and relational patterns, IRE is centered on principles like Ubuntu (collectiveness and cooperation), consensus building, and self-organization, The authors assert that Indigenous evaluators tend to emphasize the "why" and "how" of evaluation (focusing on objectives and cultural efficacy) over the "who" and "what" prized by contemporary Western models. | Indigenously Responsive Evaluation (IRE) Framework / Synthesized Indigenous Participatory M&E Framework: A framework developed through synthesizing data on Indigenous relational evaluation patterns, It is guided by core communal values and relational ethics, intended to enhance contemporary evaluation by drawing on local knowledge systems, | The core philosophy is the African-based Relational Paradigm, encompassing the Ubuntu ideal ("I am because we are"), which promotes collectiveness and social relationships. The approach aligns with the "Made in Africa" (MAE) evaluation mantra and incorporates principles from Culturally Responsive Indigenous Evaluation (CRIE), | Boadu and Ile (2023) Evaluation innovation in Africa: Towards indigenously responsive evaluation (IRE) philosophies, methods and practices in Ghana  The goal is to identify evaluative concepts embedded in cultural philosophies and relational patterns, which include community gatherings, traditional durbars, family groupings, and local social networks. The proposed methodology utilizes a strength-based approach to blend Indigenous philosophies with contemporary evaluation paradigms |
| Easton (2012) [41]Identifying the evaluative impulse in local culture: Insights from West African proverbs.  (Peer-reviewed paper) | West African cultures (Hausa, Bambara, Malinke, Wolof, Igbo) | The paper presents a conceptual exploration using proverbs and a case example. It demonstrates how local proverbs express evaluation-relevant perspectives on measurement, causality, and collective effort. Eliciting proverbs helps embody a mind-set and establish a climate for "stockholder" buy-in. | Proverb-Based Evaluation Approach (Implicit framework). It functions by uncovering the "evaluative impulse" embedded within local culture, classifying proverbial wisdom into key categories pertinent to evaluation (such as assessment, causal analysis, and collective effort) to foster unprecedented stakeholder buy-in and local ownership. | West African relational culture. | Use of proverbs to design methods; participatory process enabling participants to assess levels, debate factors, and make recommendations. The five categories of evaluation-relevant expressions are:  1. Assessment, Measurement, and Performance.  2. Inquiry, Causal Analysis, and Discernment.  3. Transparency, Responsibility, and Governance.  4. Stakeholder Involvement, Collective Effort, and Political Discretion  5. Planning, Foresight, and Capacity Building. |
| Bowman et al (2015) [42]Culturally responsive indigenous evaluation: A Practical Approach for Evaluating Indigenous Projects in Tribal Reservation Contexts  (Book Chapter) | Tribal Reservation Contexts (US Indian Country) | The book chapter discuses applying Culturally Responsive Indigenous Evaluation (CRIE) in Tribal contexts. CRIE requires evaluators to honor Tribal policy/sovereignty and co-create culturally relevant plans using existing data, guided by the "seven-generations" teachings. | The chapter discusses CRIE, a cultural and contextual framework. It presents 7 steps to decolonialise evaluations; involve knowledge councils and elders; integrate traditional knowledge and institutions; offer traditional gifts; ensure Tribal approval and data ownership; use findings to improve services; conduct external reviews to prevent racism and colonial practices; and empower communities by honouring knowledge, ensuring usefulness, and securing control of data. | It is grounded in tribal sovereignty and the assertion of Indigenous sovereignty is tied directly to the reclamation of Indigenous ways of knowing. It is guided by seven-generations teachings, a widely accepted Indigenous cultural understanding, framed as a sustainability theory based upon ancient epistemology, which dictates that current actions and decisions must positively affect the livelihood of all future generations (including humans, animals, and plants) and honor the past | The approaches focus on consensus-based decision-making including identification of existing data sources (rather than imposing new ones), sharing knowledge through oral methods (talking circles) and visual formats (GIS mapping). |
| Kawakami et al (2007) [43]Improving the Practice of Evaluation Through Indigenous Values and Methods: Decolonizing Evaluation Practice—Returning the Gaze From Hawaiÿi and Aotearoa  (Book Chapter) | Case study description and comparison with mainstream valuation in Kanaka Maoli (Hawaiian) communities | The chapter discusses improving evaluation using Indigenous values and cultural competence. It describes the Hoÿowaiwai Nä Kamaliÿi project, designed to empower communities through data use to determine priorities for Native Hawaiian children. | A Conceptual Framework for Indigenous Evaluation Practice defines purpose and goals set by the community agenda, seeking positive community impacts. | Kanaka Maoli/Native Hawaiian values and Indigenous epistemology are centered on identity and relationality, emphasizing the essential connectedness of individuals to their land (‘āina), their family and ancestors (whānau/‘ohana), and their language, while valuing proper relationships with spiritual power inherent in every location. | The approach presents findings through narratives, mo’olelo (stories), relationships, photos, DVDs, CDs, and videos, with conclusions shared among the project, community, evaluator, and funder, while data collection accounted for community wariness stemming from past failures. |
| Smith (2012) [44]Decolonizing Methodologies: Research and Indigenous Peoples  (Book Chapters 9, 10, 11) | Primarily focused on the experiences and political struggles of Māori (Indigenous people of Aotearoa/New Zealand), specifically addressing the history of research that shaped negative attitudes towards it. | This work discusses the profound shift required when the "researched become the researchers". Research is critiqued as being deeply implicated in the colonization process because it is concerned with defining "legitimate knowledge". The concept of Kaupapa Māori (Māori way) is presented as a mechanism for structuring research assumptions, values, and priorities, rooted in the inherent legitimacy of Māori language and culture. The methodology is designed to dismantle structures of oppression and allow Indigenous people to be seen as fully human. | Kaupapa Māori Research (KMR): A counter-hegemonic practice centered on Māori philosophy and principles and the legitimacy of Māori culture. It connects directly to the struggle for self-determination (Tino Rangatiratanga). KMR uses principles (such as Kia tūpato—be cautious/culturally safe) and models (like the tiaki or mentoring model, whangai or adoption model, power sharing model, and empowering outcomes model) to guide culturally appropriate engagement. | Decolonization Theory (Decolonization is about restoring power and humanity, not merely a metaphor or civil rights project). The philosophy is anti-colonial, aiming to replace colonial methods and philosophies with Indigenous teachings and culture. It is framed within the Indigenous Research Paradigm, emphasizing relational accountability and critique of White supremacy woven into Western knowledge creation | Kaupapa Māori Evaluation/Research focuses on activist solidarity and transformative praxis. It requires mandatory critical reflexivity by the researcher regarding their colonial lens, privilege, and biases. The ethical approach emphasises respect, generosity (Manaaki ki te tangata), listening (Titiro, whakarongo… korero), and caution (Kia tūpato). Methods are employed that align with Māori cultural protocols and traditions, such as storytelling. |
| Hood et al. (2015) [45]Culturally Responsive Evaluation: Theory, Practice, and Future Implications  (Book chapter) | Conceptual synthesis; outlines nine procedural stages of CRE  International, Indigenous, and Minoritized contexts (General/Historical) | Culturally Responsive Evaluation (CRE) builds theory on the ground by operationalizing general principles across nine procedural stages. CRE is rooted in a sense of social responsibility to the community served. | Culturally Responsive Evaluation (CRE) Framework: Nine procedural stages: (1) preparing for the evaluation, (2) Engage stakeholders, (3) Identify evaluation purpose(s), (4) Frame the right questions, (5) Design the evaluation, (6) Select and adapt instrumentation, (7) Collect the data recognising "self-as-instrument", (8) Analyze the data through valid contextualized meaning, and (9) Disseminate and use the results through culturally appropriate and respectful. | Social Responsibility; Historical context of African American evaluation pioneers. Congruent with Kaupapa Māori relational principles. | It suggests the use of a Culture Checklist to prompt reflection |
| Cram et al. (2015) [46]Transformative and indigenous frameworks for multimethod and mixed methods research.  (Book Chapter) | Māori (Aotearoa/New Zealand) | The chapter discusses aligning Transformative and Indigenous frameworks for mixed methods research (MMR), emphasising social justice, relationships, and the use of MMR to dismantle inequalities. | The Transformative Paradigm (TP), a research worldview where social justice operates as the first principle, having emerged to address the everyday realities and aspirations of marginalised people by prioritizing the analysis of power inequities and the impact of privilege to achieve transformative social change. TP  and indigenous frameworks in MMR should be used to advance social justice and eliminate inequalities. | Transformative ontological assumption (reality shaped by values) and Indigenous epistemology (knowledge is relationships). | Mixed methods cyclical design. Use of traditional methods (talk-story) alongside contemporary methods. Reciprocity facilitated by kinship connections (whakapapa). |
| Eakins et al (2023) [47]Indigenous evaluation toolkit: An Actionable Guide for Organizations Serving American Indian/Alaska Native Communities through Opioid Prevention Programming  (Grey Literature) | Primarily targets Tribal and Urban Indian public health programs in the US. It is specifically focused on addressing the opioid overdose crisis and related prevention, treatment, and recovery support services. | An actionable guide to implementing Indigenous Evaluation (IE) approaches by translating the foundational Indigenous Evaluation Framework (IEF) into practical steps for health promotion and prevention efforts. The guide frames Indigenous evaluation as a continuous learning process (or "river journey" metaphor) that is interwoven with program design and implementation. It empowers Indigenous communities to tell their own stories and reclaim Indigenous ways of knowing. The goal is to improve community health and wellness by centering cultural knowledge systems and holistic healing approaches | Indigenous Evaluation Toolkit (IET), based on the Indigenous Evaluation Framework (IEF) (LaFrance & Nichols, 2009). The IEF organizes evaluation around five core values: Indigenous Knowledge, People of a Place, Centrality of Community and Family, Honoring our Gifts (Personal Sovereignty), and Tribal Sovereignty | Rooted in the Indigenous Research Paradigm and the philosophy that knowledge is holistic, interconnected, and spiritual. The goal is decolonizing data—reclaiming data production for Indigenous people, by Indigenous people. It uses Indigenous worldviews (epistemologies) as the basis for determining merit and worth | Culturally Responsive Indigenous Evaluation (CRIE). Emphasizes Community Participatory Approaches (CBPR) and continuous reflection/learning. Requires intentional self-reflection and reflexivity from evaluators regarding their own biases and relationship with colonization. Adheres to Tribal Data Sovereignty principles (Ownership, Control, Access, Possession). The guide encourages the integration of Indigenous and Western methods through "Two-Eyed Seeing". Methods prioritize Indigenous knowledge sources such as storytelling, sharing testimony, talking circles, and culturally specific methods over traditional interviews or focus groups |
| Australian Government Productivity Commission (2020) [48]A Guide to Evaluation under the Indigenous Evaluation Strategy  (Grey Literature) | Australian Government agencies evaluating both Indigenous-specific and mainstream policies and programs that affect Aboriginal and Torres Strait Islander people. | Practical advice for Australian Government agencies on implementing the principles and governance arrangements of the Indigenous Evaluation Strategy (IES). This guide is a companion to the overarching IES, providing direction to program managers and commissioners on how to center Aboriginal and Torres Strait Islander people, perspectives, priorities, and knowledges at every stage of the evaluation process. It emphasizes that evaluation must be integrated early into policy design and that Aboriginal and Torres Strait Islander people must shape evaluation questions. | Indigenous Evaluation Strategy (IES) Guiding Principles: This framework is underpinned by one overarching principle, Centring Aboriginal and Torres Strait Islander people, perspectives, priorities and knowledges, and four core principles: Credible, Useful, Ethical, and Transparent. The core principle of "Credible" is interpreted through the lens of cultural responsiveness, incorporating Aboriginal and Torres Strait Islander values and knowledges alongside rigorous methodology | The Strategy recognizes the strengths and diversity of Aboriginal and Torres Strait Islander people, communities, knowledges, histories, and cultures. It supports Aboriginal and Torres Strait Islander people's right to self-determination and active participation in decision-making. Ethical practice is guided by principles such as yindyamarra, which emphasizes acting with honor, respect, wisdom, and responsibility | Culturally Safe and Culturally Responsive practice is mandatory. The evaluation process is holistic and iterative, requiring continuous planning and integration. It requires Aboriginal and Torres Strait Islander leadership and/or representation on governance or steering committees. Evaluators (especially non-Indigenous) must demonstrate cultural capability and address their own biases and White privilege. The guide encourages the use of a combination of evaluation types, approaches, and methods—including qualitative and quantitative, and Western and Indigenous methods. |
| Kwok and Faivel (2022) [49]Indigenous evaluation: how you do it is as important as what you find out  (Grey Literature) | Australia, Western Desert of the Pilbara, focusing on the Martu people and the community development organization Kanyirninpa Jukurrpa (KJ). | This article documents the methodology for evaluating KJ's long-term impact, emphasizing that the evaluation must be Martu-led to capture their genuine experiences. The methodology was designed to be highly flexible and centered on Martu-defined outcomes. The successful process led to a sense of community ownership and revealed a holistic Martu worldview where outcomes are interconnected, providing important lessons for evaluators and policy makers | Martu-led Evaluation: A practical approach where the design, criteria, and consultation methods are determined by the Martu community, prioritizing authentic Martu assessments over imposing conventional quantitative methods. The framework aimed to tell a Martu story, by Martu, for Martu and others | The approach is driven by the principle of Indigenous self-determination and the need to reflect a holistic Martu worldview. It critiques evaluation practices that quantify impact at the expense of authenticity | Indigenous-led/Martu-led approach requires an adaptive methodology. It requires evaluators to adopt a mindset of flexibility, empathy, and critical reflection. Key principles include relational accountability (demonstrated by long-term partnership and trust). Methods involved co-designing 11 outcomes valued by Martu and adapting data collection from rigid voting to informal, conversational methods (e.g., during community activities like barbecues. |
| Institute of Education Science (2021)[50] What are current Indigenous approaches to education research and evaluation, and what are some examples of them in use?  (Grey Literature) | Focuses on education research and evaluation within various Indigenous contexts, particularly those served by the Regional Educational Laboratories (RELs), including Indigenous cultures in the REL Pacific region (Hawaiʻi, Marshall Islands) and North American Indigenous communities (Native American Tribal Nations). | This guide positions Indigenous methodologies as approaches that situate research within the context of the data source(s), meaning no single Indigenous method is universal. It groups approaches by Indigenous cultures and provides examples of frameworks and methods that value local knowledge, such as the Evaluation with Aloha Framework (Hawaiʻi) and Kaupapa Māori (Aotearoa/New Zealand). It highlights the importance of using Indigenous worldviews to inform the collection and use of data | Culturally Responsive Indigenous Evaluation (CRIE): An approach that uses both traditional knowledge and contemporary Indigenous theory to design and implement evaluations within Tribal Nations. Indigenous Evaluation Model/Framework (IEF): Frames the process of conducting evaluations within tribal communities' core cultural values and knowledge | Rooted in the Indigenous Research Paradigm and the philosophical recognition that Indigenous methodologies value the worldview, perspectives, values, and lived experiences of the community. The approach is linked to honoring Indigenous sovereignty | Methodologies must situate research within context, emphasizing local constructs, methods, and theories derived from local experiences. Two-Eyed Seeing is suggested for connecting Western and Indigenous approaches. Also includes Culturally Responsive Indigenous Evaluation (CRIE). Methods cited include talking circles (to promote respect through protocols, valuing, and sharing lived experiences), Collaborative Autoethnography (CAE), storytelling (Moʻolelo), and the use of culturally rooted metaphors. The approach emphasizes Two-Eyed Seeing to connect Western and Indigenous approaches for responsible research. |
| National Collaborating Centre for Aboriginal Health (2013) [51]Indigenous Approaches to Program Evaluation  (Grey Literature) | Indigenous/Aboriginal Contexts (General) | This resource discusses culturally sensitive program evaluation and Indigenous knowledge generation.  Program evaluation should focus on improving programming based on participant needs and include crucial contextual factors (historical, social, cultural, and environmental). | The implicit framework described requires evaluators to adhere to a code of conduct based on cultural protocols (such as acting with respect, reciprocity, and responsibility) and position themselves within a relationship of trust and respect with the community. | Mainly grounded in cultural relevance and contextual factors. | Focus on generating Indigenous knowledge, methodologies, and participatory frameworks. |
| Government of Canada (2022) [52]Reflecting on Indigenous Evaluation Frameworks by Gladys Rowe (Appendix C)  (Grey Literature) | Canadian Indigenous peoples (Victim Services and Supports) | The resource discusses indigenous evaluation as resistance and resurgence. Indigenous evaluation is an act of resistance/resurgence, creating space for Indigenous ways of knowing. Evaluation is defined as a personal process of deep reflection and contemplation (not externally applied indicators). | Indigenous Evaluation Framework/Bundle: Provides opportunities to evaluate based on meaningful measures of success derived from Indigenous values. | The theoretical underpinning of this paper is framed by resurgence and Indigenous ways of knowing, being, feeling, and doing, guided by the OCAP principles of ownership, control, access, and possession. | Deep reflection and contemplation (personal process). Ceremony should be an important element of the process. |
| Bremner et al (2020) [53]Indigenous approaches to evaluation and research – Exploring Indigenous Approaches to Evaluation and Research in the Context of Victim Services and Supports  (Grey Literature) | Canada, focusing on developing appropriate evaluation mechanisms for Indigenous-specific victim programs and services. | Focuses on shifting evaluation from imposing external indicators to a process of deep reflection and contemplation (the closest Indigenous definition in Cree).  Indigenous evaluation (IE) is an act of resistance and resurgence linked to Indigenous rights to self-determination and self-governance. It requires evaluators to challenge the privileging of Eurocentric values. The methodology shifts focus from fulfilling funder requirements to being a continuous learning process where people reflect on what worked and determine the path ahead. The ultimate goal is to effect systemic change by providing evidence that supports Indigenous ways of working | Indigenous Evaluation Framework (IEF): The conceptual foundations are based on IEF principles, emphasizing that evaluation must be holistic and contextualized to a specific place, time, community, and history. It centers Relational Accountability—demonstrating respect, responsibility, and reciprocity throughout the process | Rooted in the Indigenous Research Paradigm and Indigenous worldviews. The ontology is fundamentally relational, emphasizing connection to the land, spiritual realm, and each other. The approach is driven by an explicit decolonization intent | Culturally Responsive Evaluation (CRE). Utilises strengths-based perspectives to focus on resilience and assets rather than deficits. Requires a Trauma-informed approach to acknowledge historical and intergenerational trauma. Requires adherence to the OCAP principles (Ownership, Control, Access, Possession) for data governance and ethics. Evaluators must engage in mandatory critical self-reflection/reflexivity regarding their positionality, biases, and relationship with colonization.  Methods integrate Indigenous approaches like storytelling (as a fundamental method for sharing knowledge), ceremony (essential for knowledge gathering), and the use of culturally rooted metaphors. |
| Native Plants and Foods Curriculum Portal (N.D.) [54]Indigenous Evaluation  (Grey Literature) | Designed to support educators incorporating Indigenous plant and food curricula. Context focuses on Coast Salish communities and promotes traditional foods and medicines for health and wellness | A guide to using culturally appropriate and engaging methods for evaluating the impact of Indigenous plant and food curricula. Indigenous evaluation is framed as a long-standing practice where Native people have always collected data for community benefit. The evaluation process is guided by four core values emphasizing resilience, decolonizing data, and centering the community. The curriculum is intended to increase knowledge and skill, sense of community, and sense of season and place. It stresses the need for transparency with students regarding how their reflections will be used or shared | Indigenous Evaluation (IE) approach rooted in the four core principles of the Urban Indian Health Institute’s (UIHI) Indigenous Evaluation Framework: Community is created wherever Native people are, Resilient and strength-based, Decolonizing data, and Centering of the community. The tool also uses a Visual Logic Model | The approach is grounded in the recognition that Indigenous knowledge, practices, and values are valid and should be prioritized over Western approaches when telling the community's story. The goal is to collect rigorous data with the intent to benefit urban Indian communities and reclaim data | Indigenous Evaluation emphasise on indigenous ways of knowing (stories, art, conversations), participatory evaluation, and self-reflection. The process requires Indigenous culture to be centered at all levels, including evaluation. Methods used include stories, art, and conversations as equally valid as Western methods like surveys and interviews. Dissemination uses a Story Map (a visual tool) to share knowledge. Evaluation uses a modified Visual Logic Model that coalesces Cascadia region Indigenous values and environmental sustainability learning outcomes |
| Polansky and  Echo-Hawk (2021) [55]Building the Sacred: An Indigenous Evaluation Framework for Programs Serving Native Survivors of Violence  (Grey Literature) | Native survivors of violence from American Indian and Alaska Native communities in the United States. | The Indigenous Evaluation Framework for programs serving Native survivors of violence was built on resilience and strength, prioritizing storytelling to capture the quality of care and connection. Mission is to decolonize data, for Indigenous people, by Indigenous people. | Building the Sacred: An Indigenous Evaluation Framework identifies four "places" of existing Indigenous evaluation practice—walking with relatives, planting seeds of strength, healing, and adapting in community—protecting space for grief and healing—to ensure evaluation results are communicated as meaningful stories that uplift Indigenous cultures rather than reinforcing external, restrictive funding requirements. | The theoretical underpinning of this paper is grounded in decolonisation, a strength-based approach, and the Urban Indian Health Institute (UIHI) Indigenous Evaluation Framework. | The approach suggests the use of observation as a key data source, trauma-informed monitoring and evaluation with attuned surveys and visual logic models, and a collaborative adaptation process involving monthly reflection, storytelling, and discussion, alongside inductive thematic analysis. |
| Aman et al (2022) [56]Indigenous Evaluation 101 Guidebook  (Grey Literature) | Primarily targets Tribal and indigenous communities in Minnesota, the United States. | An actionable guide for programs to implement Indigenous Evaluation (IE) approaches for health promotion and prevention efforts by translating the Indigenous Evaluation Framework (IEF) into an iterative, continuous learning cycle over four phases and ten steps (Envision & Design, Embark, Assess Progress, Celebrate & Act). Indigenous evaluation is framed as a long-relied-upon practice of community review and reflection. The Toolkit aims to empower Indigenous communities to tell their own stories and prioritize Indigenous ways of knowing. It requires evaluators to honour Tribal sovereignty and co-create culturally relevant plans guided by the philosophy that evaluation is a continuous learning process focused on healing and wellness | Indigenous Evaluation Framework (IEF) serves as the conceptual foundation, structuring the evaluation around five core values: indigenous knowledge, people of a place, centrality of community and family, honouring our gifts (personal sovereignty), and tribal sovereignty | Indigenous Research Paradigm / Tribal Critical Race Theory (TCT). The approach is based on Indigenous ways of knowing (epistemologies), where knowledge is holistic, interconnected, and spiritual. The fundamental goal is decolonizing data—reclaiming data production for Indigenous people, by Indigenous people | Culturally Responsive Indigenous Evaluation (CRIE). Community Participatory Approaches (CBPR). Holistic and Strengths-Based programming. Mandatory Reflective Practice for evaluators concerning positionality and power dynamics. Evaluation must adhere to principles like Tribal Data Sovereignty and adherence to Tribal IRB processes.  It incorporates Indigenous methodologies like Storytelling, ceremonial practice, and non-Western knowledge sources (Traditional, Revealed, and Empirical Knowledge). It advocates for Two-Eyed Seeing to blend Indigenous and mainstream knowledge systems. |
| The Great Plains Tribal Leaders’ Health Board (2022) [57]Indigenous Evaluation Toolkit  (Grey Literature) | Developed for Tribal and indigenous communities in the United States | The Toolkit helps Indigenous communities tell their own stories and reclaim Indigenous ways of knowing (epistemologies) to ensure that evaluation efforts are holistic, community-centered, and continuous. It reframes impact evaluation as a continuous learning process focused on structural change and healing, rather than just objective measurement | Indigenous Evaluation Toolkit (IET) provides resources and guides evaluation from design through implementation and adaptation | Grounded in the goal of decolonizing data ("for Indigenous people, by Indigenous people"), and incorporating Tribal Critical Theory (TCT) principles | Approaches prioritise Indigenous knowledge sources such as traditional knowledge, revealed knowledge (dreams/ceremony), or feelings about community wellbeing, collected via Indigenous methodologies like storytelling, sharing testimony, and art-based activities. |
| Indigenous Data Toolkit (2024) [58]Indigenous Approaches to Data and Evaluation Reading List  (Grey Literature) | General Indigenous Data/Evaluation | It lists foundational concepts and authors related to Indigenous data sovereignty, methodology, and co-learning. | N/A | Indigenous Data Sovereignty. | Two-Eyed Seeing (co-learning journey of weaving Indigenous and mainstream knowledges); Conversation Method. |
| Van Bavel (2022) [59]Indigenous Evaluation Methodologies  (Grey Literature) | This resource was prepared for the Community of Practice dedicated to Addressing Youth Dating Violence among indigenous communities in Canada. | This guide positions Indigenous Evaluation Methodology (IEM) as a culturally respectful practice where the program is fundamentally inseparable from its context. It defines decolonization as the process of removing colonial elements that invalidate Indigenous knowledge. The paper emphasizes that researchers must build relationships with the community to prevent "pan-Indigenizing" and ensure local traditions inform analysis | The resource discusses Indigenous Evaluation Methodology (IEM), a framework emphasizing that knowledge is developed within a specific context, which must be acknowledged. Its core values include Relationships and Collaboration. It also highlights Two-Eyed Seeing as a critical principle for weaving together the strengths of Western knowledge and Indigenous ways of knowing, ensuring the outcome is rigorous and beneficial to the community | Indigenous Research Paradigm where knowledge is broadly rooted in an Indigenous understanding of the relationship to the land, the spiritual realm, and each other. The approach is driven by the intent of decolonization and the principle of self-determination for Indigenous communities. It aligns with Tribal Critical Race Theory (TCT) principles | Culturally Responsive, contextual, and trauma-informed Practice: Evaluators must be intentional when selecting designs and methods and should ensure the process adheres to principles like OCAP (Ownership, Control, Access, Possession). The evaluator is required to engage in self-reflection and reflexivity regarding power dynamics. |
| Christian aid. 2022 [60] Decolonising Evaluation - Whose Value Counts?  (Grey Literature) | International Development/INGO (Global North and South) | The resource advocates for shifting evaluation practice toward a decolonial approach where decoloniality is the rebuilding of practice in new directions. Advocates for flexibility in accountability to prioritize local community ways of knowing over compliance. | Decolonial Approach: Guided by Intentionality, Participant-led processes, and Culture Shift. | Decolonisation/Decoloniality; challenge to Western conceptualised rationality. | Focus on people-centered stories over quantifiable data. Evaluators should move beyond rigid requirements and shift their role from expert to local supporter. |

## References

1.	Johnson P, Dancey SJ, Wonneck LA, Johnson PC. Kwayâtisiwin (“We Prepare”): Understanding the Methods of Preparation for Evaluating Nêhiyawak-Based Research. Canadian journal of program evaluation. 2025;39(3):509-25. doi: 10.3138/cjpe-2025-0007.

2.	Seneca CN, McCarn BM, Baker L. Responsibility to All Relations: Indigenous Evaluation of an AI/AN Public Health Workforce Development Program. Canadian journal of program evaluation. 2025;39(3):526-46. doi: 10.3138/cjpe-2025-0008.

3.	Thiessen S, Chouinard JA. Recognizing the Need for a Trauma-Informed Approach in the Evaluation of an Indigenous Program in British Columbia. Canadian journal of program evaluation. 2025;39(3):571-84. doi: 10.3138/cjpe-2024-0020.

4.	Dazzo GP. Evaluation as Reparation, Reclamation, and Renewal: Reorienting Our Justice Signposts Toward Restorative Validity. The American journal of evaluation. 2025. doi: 10.1177/10982140251350093.

5.	Erb T, Garcia J, Stelkia K. Relational Systems and the Principles of Self-Determination, Reciprocity, and Mutual Benefit: Enacting Indigenous Ways of Knowing in the Evaluation of an Indigenous Health Research Network in the Province of British Columbia, Canada. Canadian journal of program evaluation. 2025;39(3):487-508. doi: 10.3138/cjpe-2024-0036.

6.	van der Puije N. Decolonising Evaluation Practice in International Development Cooperation Through an African Religion Lens. Religions (Basel, Switzerland ). 2025;16(5):609. doi: 10.3390/rel16050609.

7.	Puije NVD, Satzinger F. Decolonising international development evaluation: rethinking OECD DAC evaluation criteria from a decolonial perspective*. Development in Practice. 2025:1-15. doi: 10.1080/09614524.2025.2482780.

8.	Sehgal A, Henderson R, Murry A, Crowshoe L, Barnabe C. Advancing health equity for Indigenous peoples in Canada: development of a patient complexity assessment framework. BMC primary care. 2024;25(1):144-15. doi: 10.1186/s12875-024-02362-z.

9.	Stefanski C, Abonyi DS, Katz DR, Nicholls DR, Kewistep G, Goodridge DD, et al. Indigenous evaluation: A scoping review of Indigenous evaluation methods and guiding principles. Turtle Island Journal of Indigenous Health. 2025;1(4). doi: 10.33137/tijih.v1i4.41128.

10.	Gordon HSJ, Ayoub C, Grant J, Blocklin M. Engaging Indigenous Populations in Evaluation through Indigenous Methodologies. Washington, DC: OPRE, 2024.

11.	Dutta MJ, Kaur-Gill S, Metuamate S. Decolonizing Impact Through the Culture-Centered Approach to Health Communication: Mobilizing Communities to Transform the Structural Determinants of Health. Health communication. 2024;39(14):3581-9. doi: 10.1080/10410236.2024.2343466.

12.	Melro CM, Landry J, Matheson K. A scoping review of frameworks utilized in the design and evaluation of courses in health professional programs to address the role of historical and ongoing colonialism in the health outcomes of Indigenous Peoples. Advances in health sciences education : theory and practice. 2023;28(4):1311-31. doi: 10.1007/s10459-023-10217-y.

13.	Povey J, Raphiphatthana B, Torok M, Nagel T, Mills PPJR, Sells JRH, et al. An emerging framework for digital mental health design with Indigenous young people: a scoping review of the involvement of Indigenous young people in the design and evaluation of digital mental health interventions. Systematic reviews. 2023;12(1):108-19. doi: 10.1186/s13643-023-02262-w.

14.	Sutthibut K, Itharat A, Singchungchai P, Wanichsetakul P, Pipatrattanaseree W, Ooraikul B, et al. Development of an Assessment Tool of Menstrual-Cycle-Related Signs and Symptoms Based on Thai Traditional Medicine Principles for Evaluation of Women’s Health. Evidence-based complementary and alternative medicine. 2021;2021(NA):1-16. doi: 10.1155/2021/9977773.

15.	Bailie J, Laycock AF, Peiris D, Bainbridge RG, Matthews V, Cunningham FC, et al. Using developmental evaluation to enhance continuous reflection, learning and adaptation of an innovation platform in Australian Indigenous primary healthcare. Health research policy and systems. 2020;18(1):45-11. doi: 10.1186/s12961-020-00562-4.

16.	Lawrence TJ, James RD. Good Health and Wellness: Measuring Impact Through an Indigenous Lens. Preventing chronic disease. 2019;16:E108. doi: 10.5888/pcd16.180655.

17.	Cargo M, Potaka-Osborne G, Cvitanovic L, Warner L, Clarke S, Judd J, et al. Strategies to support culturally safe health and wellbeing evaluations in Indigenous settings in Australia and New Zealand: a concept mapping study. International journal for equity in health. 2019;18(1):194-17. doi: 10.1186/s12939-019-1094-z.

18.	Rarere M, Oetzel J, Masters-Awatere B, Scott N, Wihapi R, Manuel C, et al. Critical reflection for researcher–community partnership effectiveness: the He Pikinga Waiora process evaluation tool guiding the implementation of chronic condition interventions in Indigenous communities. Australian journal of primary health. 2019;25(5):478. doi: 10.1071/PY19022.

19.	LaFrance J, Nichols R. Reframing Evaluation: Defining an Indigenous Evaluation Framework. Canadian journal of program evaluation. 2008;23(2):13-31. doi: 10.3138/cjpe.23.003.

20.	Whitau R, Ockerby H. Yarning with the Stars Project: An Indigenous evaluation protocol for a sport for development and peace program. Journal of sport for development. 2019;7(13).

21.	Chilisa B, Major TE, Gaotlhobogwe M, Mokgolodi H. Decolonizing and Indigenizing Evaluation Practice in Africa: Toward African Relational Evaluation Approaches. Canadian journal of program evaluation. 2016;30(3):313-28. doi: 10.3138/cjpe.30.3.05.

22.	Chilisa B, Mertens DM. Indigenous Made in Africa Evaluation Frameworks: Addressing Epistemic Violence and Contributing to Social Transformation. The American journal of evaluation. 2021;42(2):241-53. doi: 10.1177/1098214020948601.

23.	Chilisa B, Major TE, Khudu-Petersen K. Community engagement with a postcolonial, African-based relational paradigm. Qualitative research : QR. 2017;17(3):326-39. doi: 10.1177/1468794117696176.

24.	Dlakavu A, Mathebula J, Mkhize S. Decolonising and indigenising evaluation practice in Africa: Roadmap for mainstreaming the Made in Africa Evaluation approach. African evaluation journal. 2022;10(1):1-10. doi: 10.4102/aej.v10i1.620.

25.	Kelly LM, Htwe PPT. Decolonizing Community Development Evaluation in Rakhine State, Myanmar. The American journal of evaluation. 2024;45(1):68-85. doi: 10.1177/10982140221146140.

26.	Brown MA, Di Lallo S. Talking Circles: A Culturally Responsive Evaluation Practice. The American journal of evaluation. 2020;41(3):367-83. doi: 10.1177/1098214019899164.

27.	Pant I, Khosla S, Lama JT, Shanker V, AlKhaldi M, El-Basuoni A, et al. Decolonising global health evaluation: Synthesis from a scoping review. PLOS global public health. 2022;2(11):e0000306-e. doi: 10.1371/journal.pgph.0000306.

28.	Renmans D, Sarkar N, Van Belle S, Affun‐Adegbulu C, Marchal B, Mukumbang FC. Realist evaluation in times of decolonising global health. The International journal of health planning and management. 2022;37(S1):37-44. doi: 10.1002/hpm.3530.

29.	Igwe PA, Madichie NO, Rugara DG. Decolonising research approaches towards non-extractive research. Qualitative market research. 2022;25(4):453-68. doi: 10.1108/QMR-11-2021-0135.

30.	Zwiener-Collins N, Jafri J, Saini R, Poulter T. Decolonising quantitative research methods pedagogy: Teaching contemporary politics to challenge hierarchies from data. Politics (Manchester, England). 2023;43(1):122-38. doi: 10.1177/02633957211041449.

31.	Dighe S, Matthias C. Deconstructing the Imperial Episteme: Decolonizing Knowledge Production in Program Evaluation. Journal of multidisciplinary evaluation. 2023;19(44). doi: 10.56645/jmde.v19i44.781.

32.	Goldman I, Morkel C, Agbodjan ED, Molaiwa TG. Decolonising national evaluation systems. African evaluation journal. 2024;12(1):1-12. doi: 10.4102/aej.v12i2.731.

33.	Goforth AN, Nichols LM, Sun J, Violante A, Christopher K, Graham N. Incorporating the indigenous evaluation framework for culturally responsive community engagement. Psychology in the schools. 2022;59(10):1984-2004. doi: 10.1002/pits.22533.

34.	Kane F, Archibald T. Ubuntu and Afrofeminism for Decolonizing Evaluation. Journal of multidisciplinary evaluation. 2023;19(44). doi: 10.56645/jmde.v19i44.793.

35.	Waapalaneexkweew. Looking Backward but Moving Forward: Honoring the Sacred and Asserting the Sovereign in Indigenous Evaluation. The American journal of evaluation. 2018;39(4):543-68. doi: 10.1177/1098214018790412.

36.	Millet H. Ukombozi Means Liberation: A Case for Decolonizing Global Health Research, Methodology, and Praxis: ProQuest Dissertations & Theses; 2019.

37.	Kerr S. Kaupapa Maori theory-based evaluation. Evaluation journal of Australasia. 2012;12(1):6-18. doi: 10.1177/1035719X1201200102.

38.	Cram F. Lessons on Decolonizing Evaluation from Kaupapa Māori Evaluation. Canadian journal of program evaluation. 2016;30(3):296-312. doi: 10.3138/cjpe.30.3.04.

39.	Boadu ES, Ile I. Beyond the Buzzword: A Framework for an Indigenous Relational Evaluation in Traditional Communities in Ghana. The American journal of evaluation. 2024;45(3):467-88. doi: 10.1177/10982140211048459.

40.	Boadu ES, Ile I. Evaluation innovation in Africa: Towards indigenously responsive evaluation (IRE) philosophies, methods and practices in Ghana. African journal of science, technology, innovation and development. 2023;15(5):624-36. doi: 10.1080/20421338.2023.2173864.

41.	Easton PB. Identifying the Evaluative Impulse in Local Culture: Insights From West African Proverbs. The American journal of evaluation. 2012;33(4):515-31. doi: 10.1177/1098214012447581.

42.	Bowman NR, Francis CD, Tyndall M. Culturally Responsive Indigenous Evaluation: A Practical Approach for Evaluating Indigenous Projects in Tribal Reservation Contexts. In: Hood S. HR, Frierson H. (Eds.),, editor. Continuing the Journey to Reposition Culture and Cultural Context

in Evaluation Theory and Practice: Information Age Publishing; 2015. p. pp. 335–59.

43.	Kawakami AJ, Aton K, Cram F, Lai MK, Porima L. Improving the practice of evaluation through indigenous values and methods: Decolonizing evaluation practice–Returning the gaze from Hawaiÿi and Aotearoa. Hulili: Multidisciplinary Research on Hawaiian Well-Being. 4: Kamehameha Schools; 2007. p. 219–42.

44.	Smith LT. Decolonizing Methodologies Research and Indigenous Peoples. 2nd ed. London: Zed Books; 2012.

45.	Hood S, Hopson RK, Kirkhart KE, Hatry HP, Newcomer KE, Wholey JS. Culturally Responsive Evaluation. Hoboken, NJ, USA: John Wiley & Sons, Inc; 2015. p. 281-317.

46.	Cram F, Mertens DM, Hesse-Biber SN, Johnson RB. Transformative and Indigenous Frameworks for Multimethod and Mixed Methods Research. Oxford Library of Psychology: Oxford University Press; 2015.

47.	Eakins D, Gaffney A, Marum C, Wangmo T, Parker M, Magarati M. Indigenous evaluation toolkit: An Actionable Guide for Organizations Serving American Indian/Alaska Native Communities through Opioid Prevention Programming. Seven Directions at the University of Washington, 2023.

48.	Productivity Commission. A Guide to Evaluation under the Indigenous Evaluation Strategy. Commonwealth of Australia, 2020.

49.	Kwok A, Faivel S. Indigenous evaluation: how you do it is as important as what you find out: Social Ventures Australia Consulting (SVA); 2022 [cited 2025 July]. Available from: <https://www.socialventures.org.au/our-impact/indigenous-evaluation-how-you-do-it-is-as-important-as-what-you-find-out/>.

50.	Institute of Education Science. What are current Indigenous approaches to education research and evaluation, and what are some examples of them in use? (Frequently Asked Questions(Regional Educational Laboratory Pacific, 2021.

51.	National Collaborating Centre for Aboriginal Health. Indigenous Approaches to Program Evaluation. 2013.

52.	Government of Canada. Appendix C – Reflecting on Indigenous Evaluation Frameworks by Gladys Rowe: Government of Canada; 2022 [cited 2025 June]. Available from: <https://www.justice.gc.ca/eng/rp-pr/jr/eiaer-eaame/appendixc-annexec.html>.

53.	Bremner LK, Johnston ALK, Rowe G, Sasakamoose J. Exploring Indigenous Approaches to Evaluation and Research in the Context of Victim Services and Supports. Government of Canada; 2020.

54.	Native Plants and Foods Curriculum Portal. Indigenous Evaluation N.D. [cited 2025 August]. Available from: <https://nativeplantsandfoodsportal.org/node/48>.

55.	Polansky L, Echo-Hawk A. Building the Sacred: An Indigenous Evaluation Framework for Programs Serving Native Survivors of Violence. Seattle, WA: Urban Indian Health Institute, 2021.

56.	Aman J, Bowman N, MartinRogers N, Taylor-Schiro E, Tokunaga J, Valorose J. Indigenous Evaluation 101 Guidebook. Minnesota: Bowman Performance Consulting and Wilder Research, 2022.

57.	Great Plains Tribal Leaders’ Health Board. Indigenous Evaluation Toolkit South Dakota: Tribal Epidemiology Center; 2022 [cited August 2025]. Available from: <https://www.greatplainstribalhealth.org/great-plains-tribal-epidemiology-center/indigenous-evaluation-toolkit-133.html>.

58.	Indigenous Data Toolkit. Indigenous Approaches to Data and Evaluation Reading List. Aotearoa (New Zealand): Indigenous Data Toolkit,, 2022.

59.	Van Bavel B. Indigenous Evaluation Methodologies. Prevnet, 2022.

60.	Backhouse J. Decolonising Evaluation - Whose Value Counts? London: Christian aid, 2022.
