# Supplementary material for: Decolonising global health evaluations: A scoping review of methodological frameworks
Source: PLOS Glob Public Health. 2026 Sep 25;6(9):e0007362. doi: 10.1371/journal.pgph.0007362 (PMC13614595; doi:10.1371/journal.pgph.0007362)
Supplement: Supplementary file 6 [file pgph.0007362.s006.docx]

Decolonising Global Health Evaluations: A Scoping Review of Methodological Frameworks

**Response to the Editor and Reviewers**

We thank the editor and reviewers for the thoughtful and constructive feedback on our manuscript, which has strengthened the paper. In the Introduction setcion, we have added working definitions. We have added an explicit accountability statement and reworked this section to make clear that we do not claim authority over Indigenous evaluation. In the results section, we have also clarified that our typology is non-linear and its categories permeable and added a summary table of the four different domains. In the discussion section, we made the implications concrete and stakeholder-specific and linked them to an applied reflexive planning guide published alongside this review; tightened the manuscript; and addressed the points of detail raised.

All changes are in track changes in the revised manuscript. We respond to each comment point by point below. Comments are summarised in the left column and our responses, with their locations in the manuscript, on the right.

| 1. Please amend your detailed Financial Disclosure statement. This is published with the article. It must therefore be completed in full sentences and contain the exact wording you wish to be published.  i. Please clarify all sources of financial support for your study. List the grants, grant numbers, and organizations that funded your study, including funding received from your institution.  ii. State the initials, alongside each funding source, of each author to receive each grant.  iii. State what role the funders took in the study. If the funders had no role in your study, please state: “The funders had no role in study design, data collection and analysis, decision to publish, or preparation of the manuscript.”  iv. If any authors received a salary from any of your funders, please state which authors and which funders. | The Funding statement has been completed. It now names the LSHTM Equity, Diversity and Inclusion (EDI) Small Grant (awarded to RP, SS and MR, 2024) and Centre for Evaluation funding (MG and LP, 2025); records that SH received a salary contribution from both grants; notes that open-access fees were met through the PLOS–LSHTM transformative agreement; and states that the funders had no role in study design, data collection and analysis, the decision to publish, or preparation of the manuscript. |
| --- | --- |
| 2. We note that your Data Availability Statement is currently as follows: The aggregated data supporting the findings of this study are available in this paper and in the appendices. Please confirm at this time whether or not your submission contains all raw data required to replicate the results of your study. Authors must share the “minimal data set” for their submission. PLOS defines the minimal data set to consist of the data required to replicate all study findings reported in the article, as well as related metadata and methods. | The statement has been revised for accuracy. It clarifies that the review generated no primary participant data, and that the full data-extraction (charting) table for all 59 included records is provided as Appendix D of the Supporting Information. It also signposts the Boolean search strings used in databases of published literature (Appendix A), grey-literature sources (Appendix B) and the PRISMA-ScR checklist (Appendix C). |
| 3. Please ensure that your Ethics Statement is available in its entirety at the beginning of your Methods section, under a subheading 'Ethics Statement'. | An Ethics Statement subheading now appears at the start of the Methods carrying the compliance text (review of published literature; no primary data or human participants; approval not required). A short reflective treatment of research ethics is retained within the reflexive section and cross-referenced to the positionality and accountability statements. |
| **Reviewer 1** | |
| Reviewer #1: This manuscript is a strong and promising scoping review on decolonial and Indigenous approaches to evaluation in global health. Its major contribution is the development of a typology of evaluation approaches that distinguishes between culturally sensitive, participatory, decolonial/anticolonial, and Indigenous evaluation methodologies. This helps clarify a field where terms are often used loosely or interchangeably.  The paper’s strengths include:  (1) A compelling opening metaphor from Achebe that frames epistemic humility.  (2) Clear research questions and a well-justified scoping review approach.  (3) Strong engagement with epistemic injustice, coloniality, Indigenous sovereignty, and relational accountability.  (4) A useful distinction between deconstruction of colonial evaluation systems and reconstruction of Indigenous/decolonial alternatives.  (5) A thoughtful reflexive section that acknowledges the limits of using scoping review methods to study Indigenous and decolonial knowledge. | Thank you for your encouraging comments and for the constructive feedback . We appreciate this. |
| The main concerns are:  (1) The paper acknowledges the absence of Indigenous scholars among the authors or advisory group, but this needs a stronger accountability response.  (2) The manuscript is too long and somewhat repetitive.  (3) The typology is helpful but needs clearer explanation so it does not appear overly linear or hierarchical.  (4) The implications for global health evaluation practice need to be made more concrete.  (5) The repeated use of “‘Euro-centric’ (sic)” becomes distracting and should be handled more cleanly. | A new Accountability statement names the contradiction at the heart of the work — a Global North team, supported by Global North funders, writing about Indigenous and decolonial evaluation without Indigenous co-authorship or community governance — and states plainly that the review “does not claim authority over Indigenous evaluation” and does not speak for these communities, but is directed at the Global North institutions whose practices the literature calls to account and who can learn from Indigenous methodologies.  We also reworked the positionality section (‘Gaze and researchers’ positionalities’) to treat positionality as an analysis of power rather than a confessional preface, and added a proportionality argument to the Implications section so that responsibility tracks power rather than falling on the least powerful.  We tightened the positionality material, reduced overlap across the four methodology subsections and the reflective ‘note on practice’, and removed redundant passages, including duplicated framing between the Implications and the Conclusion.  The typology is now explicitly described as non-linear, with permeable, frequently overlapping domains. It is presented as a spectrum of praxis (from adaptation to transformation), accompanied by a separate deconstruction/(re)construction heuristic (Figure 2), and the text states that the order is for narrative clarity only.  The Implications section now sets out stakeholder-specific actions for funders and commissioners, policymakers, educators, and practitioners and evaluators, and references concrete mechanisms and actions in table 3. It also points readers to the applied reflexive planning guide that this review informed at our centre.  Instances of ‘(sic)’ have been removed and the contested-terminology issue is instead handled through the explanatory note on terminology in the Methods. |
| Recommended decision--Major revisions.  Not because the paper is weak, but because it is important and needs more precision. The core argument is strong and very much needed: decolonial and Indigenous evaluation should not be reduced to inclusion, participation, cultural adaptation, or equity language. The deeper issue is who defines value, who controls knowledge, who interprets evidence, and who has authority over evaluation itself.  Most important revision  Add a stronger accountability statement. The authors should clarify that they are not claiming authority over Indigenous evaluation, especially given the absence of Indigenous co-authorship or advisory governance. Instead, they should position the article as a reflexive synthesis aimed at changing global health institutions, funders, researchers, and evaluators. | We explicitly added an accountability statement section in the end of the methods. |
| **Reviewer 2** | |
| Reviewer #2: Thank you for the opportunity to review this important manuscript, "Decolonising Global Health Evaluations: A Scoping Review of Methodological Frameworks." The authors have done a commendable job in addressing a timely and increasingly influential area of scholarship, and the paper makes a valuable contribution by synthesising a rapidly expanding body of literature on decolonising evaluation. It was particularly good to see the researcher positionality section. The distinction between deconstructing colonial systems and reconstructing decolonial and Indigenous-centred alternatives is particularly useful. Overall, this is a thoughtful and relevant manuscript with clear implications for global health evaluation research and practice. I have a few suggestions that may help strengthen the paper. | Thank you for the constructive feedback and thoughtful comments. |
| Introduction  The manuscript addresses an important gap in the evaluation literature. It would be helpful, however, to provide clearer definitions of key concepts such as “decolonisation,” “decolonising evaluation,” “Indigenous-centred approaches,” and “epistemic injustice” as they are used in this review. Given the diversity of interpretations across these fields, a clearer positioning of how these terms are understood would strengthen the framing. | Working definitions of the key terms (decolonising and Indigenising evaluation, and related concepts) are now provided early in the Introduction, with explicit acknowledgement that these terms are contested and diverse. |
| Methods  The scoping review methodology appears appropriate for the aims of the study. It would be helpful to include the keywords and search strings in a table for transparency. Further elaboration on how studies were grouped and how consistency in coding or interpretation was maintained across the team would also strengthen the methods section. | The full Boolean search strings are provided in Appendix A, and the grey-literature sources in Appendix B. The multi-stage search and the charting/coding process are described in the Search strategy and Analysis subsections — including a piloted charting tool, independent charting by SH and AP with reconciliation, and domains derived inductively rather than imposed a priori. Inclusion and exclusion criteria are tabulated (Table 2). |
| Results  The proposed typology is one of the strongest contributions of the paper. This section would benefit from a clearer explanation of how studies were mapped across the spectrum of evaluation praxis and how boundaries between categories were determined in practice.  A summary table outlining key characteristics of included frameworks, such as philosophical orientation, methodological features, and intended application, would also improve clarity and usability.  The distinction between deconstructive and reconstructive approaches is particularly compelling. It would be helpful to clarify where frameworks sit across this spectrum, particularly where they overlap or span both orientations. | A typology summary table now sets out, for each category, its philosophical/epistemic orientation, its position on the deconstruction–(re)construction spectrum, illustrative frameworks and methods, and its intended application and locus of epistemic authority. |
| Minor comments  Discussion  The discussion raises an important point about how decolonial language can sometimes be adopted in name rather than in substance. This section would be strengthened by including a few concrete examples of how symbolic adoption of decolonising terminology differs from deeper methodological or epistemological change.  It may also be worth engaging more directly with emerging scholarship on decolonising global health and implementation science, particularly work examining how power relations, local knowledge systems, and more equitable partnerships shape evaluation practice and evidence generation.  I also wondered whether the discussion could engage with some of the recent decolonising global health literature that has explored similar questions around power, knowledge production, and implementation. For example, Decolonizing global health: a scoping review (Mehjabeen, Patel & Jindal, 2025) and Decolonizing global health through implementation science: defining the path to equity and structural transformation (Mehjabeen, Patel & Jindal, 2026) may provide useful context for some of the arguments presented here.  While the manuscript includes a section on implications for research and practice, the recommendations would benefit from greater specificity and clearer tailoring to different stakeholder groups, including practitioners, policymakers, funders, and researchers. | We address the risk of symbolic or tokenistic use of decolonial language as distinct from substantive transformation — for example, renaming a focus group a ‘story circle’ without adopting the underlying ethical commitments — in the screening rationale and the discussion of co-optation.  As above, the Implications section now sets out stakeholder-specific actions for funders and commissioners, policymakers, educators, and practitioners and evaluators, and references concrete mechanisms and actions in table 3. It also points readers to the applied reflexive planning guide that this review informed at our centre. |
| Conclusion  The conclusion provides a clear summary of the findings. It could be strengthened by more explicitly identifying priority areas for future research, particularly work aimed at bridging the gap between decolonial theory and evaluation practice. | We have added a section on the implications at the end of the discussion section. This includes a table of implications for each of the actors. |
| Minor proofreading comments  Please review reference 57 for consistency with journal style requirements.  Please maintain consistency for abbreviation styling in line 491 and 505  A final thorough proofread is recommended to ensure consistency in terminology and formatting. | Reference 57 has been corrected and the reference list checked for consistency. Consistent abbreviations are now followed. |
