# Supplementary material for: Decolonising global health evaluations: A scoping review of methodological frameworks
Source: PLOS Glob Public Health. 2026 Sep 25;6(9):e0007362. doi: 10.1371/journal.pgph.0007362 (PMC13614595; doi:10.1371/journal.pgph.0007362)
Supplement: Peer review history

# Author comment: Author response to Decision Letter 0

**DOI:** 10.1371/journal.pgph.0007362.r001

18 Mar 2026

Attachment Submitted filename: Rebuttal letter.pdf

# Peer review: Decision Letter 0

**DOI:** 10.1371/journal.pgph.0007362.r002

**Authors:** Katrien G. Janin

23 Jun 2026

PGPH-D-26-01004

Decolonising Global Health Evaluations: A Scoping Review of methodological frameworks

PLOS Global Public Health

Dear Dr. Hafez,

Thank you for submitting your manuscript to PLOS Global Public Health. After careful consideration, we feel that it has merit but does not fully meet PLOS Global Public Health’s publication criteria as it currently stands. Therefore, we invite you to submit a revised version of the manuscript that addresses the points raised during the review process.

Your manuscript has been evaluated by two reviewers. Both reviewers indicate interest in your work, however, they also identified several concerns that need to be addressed. Please consider their comments carefully when preparing your revised manuscript and your point‑by‑point response.

Please submit your revised manuscript by Aug 21 2026 11:59PM. If you will need more time than this to complete your revisions, please reply to this message or contact the journal office at globalpubhealth@plos.org. When you're ready to submit your revision, log on to https://www.editorialmanager.com/pgph/ and select the 'Submissions Needing Revision' folder to locate your manuscript file.

Please include the following items when submitting your revised manuscript:

- A letter that responds to each point raised by the editor and reviewer(s). You should upload this letter as a separate file labeled 'Response to Reviewers'.
- A marked-up copy of your manuscript that highlights changes made to the original version. You should upload this as a separate file labeled 'Revised Manuscript with Track Changes'.
- An unmarked version of your revised paper without tracked changes. You should upload this as a separate file labeled 'Manuscript'.

Guidelines for resubmitting your figure files are available below the reviewer comments at the end of this letter.

As the corresponding author, your ORCID iD is verified in the submission system and will appear in the published article. PLOS supports the use of ORCID, and we encourage all coauthors to register for an ORCID iD and use it as well. Please encourage your coauthors to verify their ORCID iD within the submission system before final acceptance, as unverified ORCID iDs will not appear in the published article. *Only*  the individual author can complete the verification step; PLOS staff *cannot*  verify ORCID iDs on behalf of authors.

We look forward to receiving your revised manuscript.

Kind regards,

Katrien G. Janin, PhD

Staff Editor

PLOS Global Public Health

**Journal Requirements:**

1. Please amend your detailed Financial Disclosure statement. This is published with the article. It must therefore be completed in full sentences and contain the exact wording you wish to be published.

i. Please clarify all sources of financial support for your study. List the grants, grant numbers, and organizations that funded your study, including funding received from your institution. Please note that suppliers of material support, including research materials, should be recognized in the Acknowledgements section rather than in the Financial Disclosure.

ii. State the initials, alongside each funding source, of each author to receive each grant. For example: "This work was supported by the National Institutes of Health (####### to AM; ###### to CJ) and the National Science Foundation (###### to AM)."

iii. State what role the funders took in the study. If the funders had no role in your study, please state: “The funders had no role in study design, data collection and analysis, decision to publish, or preparation of the manuscript.”

iv. If any authors received a salary from any of your funders, please state which authors and which funders.

2. We note that your Data Availability Statement is currently as follows: The aggregated data supporting the findings of this study are available in this paper and in the appendices.

Please confirm at this time whether or not your submission contains all raw data required to replicate the results of your study. Authors must share the “minimal data set” for their submission. PLOS defines the minimal data set to consist of the data required to replicate all study findings reported in the article, as well as related metadata and methods (https://journals.plos.org/plosone/s/data-availability#loc-minimal-data-set-definition).

For example, authors should submit the following data:

- The values behind the means, standard deviations and other measures reported;

- The values used to build graphs;

- The points extracted from images for analysis.

Authors do not need to submit their entire data set if only a portion of the data was used in the reported study.

If your submission does not contain these data, please either upload them as Supporting Information files or deposit them to a stable, public repository and provide us with the relevant URLs, DOIs, or accession numbers. For a list of recommended repositories, please see https://journals.plos.org/plosone/s/recommended-repositories.

If there are ethical or legal restrictions on sharing a de-identified data set, please explain them in detail (e.g., data contain potentially sensitive information, data are owned by a third-party organization, etc.) and who has imposed them (e.g., an ethics committee). Please also provide contact information for a data access committee, ethics committee, or other institutional body to which data requests may be sent. If data are owned by a third party, please indicate how others may request data access.

3. Please ensure that your Ethics Statement is available in its entirety at the beginning of your Methods section, under a subheading 'Ethics Statement'.

If the reviewer comments include a recommendation to cite specific previously published works, please review and evaluate these publications to determine whether they are relevant and should be cited. There is no requirement to cite these works unless the editor has indicated otherwise.

**Additional Editor Comments (if provided):**

[Note: HTML markup is below. Please do not edit.]

Reviewers' comments:

Reviewer's Responses to Questions

**Comments to the Author**

1. Does this manuscript meet PLOS Global Public Health’s publication criteria?

Reviewer #1: Yes

Reviewer #2: Yes

**********

2. Has the statistical analysis been performed appropriately and rigorously?

Reviewer #1: N/A

Reviewer #2: N/A

**********

3. Have the authors made all data underlying the findings in their manuscript fully available (please refer to the Data Availability Statement at the start of the manuscript PDF file)?

The PLOS Data policy

Reviewer #1: Yes

Reviewer #2: Yes

**********

4. Is the manuscript presented in an intelligible fashion and written in standard English?

Reviewer #1: Yes

Reviewer #2: Yes

**********

**Reviewer #1** : This manuscript is a strong and promising scoping review on decolonial and Indigenous approaches to evaluation in global health. Its major contribution is the development of a typology of evaluation approaches that distinguishes between culturally sensitive, participatory, decolonial/anticolonial, and Indigenous evaluation methodologies. This helps clarify a field where terms are often used loosely or interchangeably.

The paper’s strengths include:

(1) A compelling opening metaphor from Achebe that frames epistemic humility.

(2) Clear research questions and a well-justified scoping review approach.

(3) Strong engagement with epistemic injustice, coloniality, Indigenous sovereignty, and relational accountability.

(4) A useful distinction between deconstruction of colonial evaluation systems and reconstruction of Indigenous/decolonial alternatives.

(5) A thoughtful reflexive section that acknowledges the limits of using scoping review methods to study Indigenous and decolonial knowledge.

The main concerns are:

(1) The paper acknowledges the absence of Indigenous scholars among the authors or advisory group, but this needs a stronger accountability response.

(2) The manuscript is too long and somewhat repetitive.

(3) The typology is helpful but needs clearer explanation so it does not appear overly linear or hierarchical.

(4) The implications for global health evaluation practice need to be made more concrete.

(5) The repeated use of “‘Euro-centric’ (sic)” becomes distracting and should be handled more cleanly.

Recommended decision--Major revisions.

Not because the paper is weak, but because it is important and needs more precision. The core argument is strong and very much needed: decolonial and Indigenous evaluation should not be reduced to inclusion, participation, cultural adaptation, or equity language. The deeper issue is who defines value, who controls knowledge, who interprets evidence, and who has authority over evaluation itself.

Most important revision

Add a stronger accountability statement. The authors should clarify that they are not claiming authority over Indigenous evaluation, especially given the absence of Indigenous co-authorship or advisory governance. Instead, they should position the article as a reflexive synthesis aimed at changing global health institutions, funders, researchers, and evaluators.

**Reviewer #2:**  Thank you for the opportunity to review this important manuscript, "Decolonising Global Health Evaluations: A Scoping Review of Methodological Frameworks." The authors have done a commendable job in addressing a timely and increasingly influential area of scholarship, and the paper makes a valuable contribution by synthesising a rapidly expanding body of literature on decolonising evaluation. It was particularly good to see the researcher positionality section. The distinction between deconstructing colonial systems and reconstructing decolonial and Indigenous-centred alternatives is particularly useful. Overall, this is a thoughtful and relevant manuscript with clear implications for global health evaluation research and practice. I have a few suggestions that may help strengthen the paper.

Major comments

Introduction

The manuscript addresses an important gap in the evaluation literature. It would be helpful, however, to provide clearer definitions of key concepts such as “decolonisation,” “decolonising evaluation,” “Indigenous-centred approaches,” and “epistemic injustice” as they are used in this review. Given the diversity of interpretations across these fields, a clearer positioning of how these terms are understood would strengthen the framing.

Methods

The scoping review methodology appears appropriate for the aims of the study. It would be helpful to include the keywords and search strings in a table for transparency. Further elaboration on how studies were grouped and how consistency in coding or interpretation was maintained across the team would also strengthen the methods section.

Results

The proposed typology is one of the strongest contributions of the paper. This section would benefit from a clearer explanation of how studies were mapped across the spectrum of evaluation praxis and how boundaries between categories were determined in practice.

A summary table outlining key characteristics of included frameworks, such as philosophical orientation, methodological features, and intended application, would also improve clarity and usability.

The distinction between deconstructive and reconstructive approaches is particularly compelling. It would be helpful to clarify where frameworks sit across this spectrum, particularly where they overlap or span both orientations.

Minor comments

Discussion

The discussion raises an important point about how decolonial language can sometimes be adopted in name rather than in substance. This section would be strengthened by including a few concrete examples of how symbolic adoption of decolonising terminology differs from deeper methodological or epistemological change.

It may also be worth engaging more directly with emerging scholarship on decolonising global health and implementation science, particularly work examining how power relations, local knowledge systems, and more equitable partnerships shape evaluation practice and evidence generation.

I also wondered whether the discussion could engage with some of the recent decolonising global health literature that has explored similar questions around power, knowledge production, and implementation. For example, Decolonizing global health: a scoping review (Mehjabeen, Patel & Jindal, 2025) and Decolonizing global health through implementation science: defining the path to equity and structural transformation (Mehjabeen, Patel & Jindal, 2026) may provide useful context for some of the arguments presented here.

While the manuscript includes a section on implications for research and practice, the recommendations would benefit from greater specificity and clearer tailoring to different stakeholder groups, including practitioners, policymakers, funders, and researchers.

Conclusion

The conclusion provides a clear summary of the findings. It could be strengthened by more explicitly identifying priority areas for future research, particularly work aimed at bridging the gap between decolonial theory and evaluation practice.

Minor proofreading comments

Please review reference 57 for consistency with journal style requirements.

Please maintain consistency for abbreviation styling in line 491 and 505

A final thorough proofread is recommended to ensure consistency in terminology and formatting.

**********

what does this mean?). If published, this will include your full peer review and any attached files.

**Do you want your identity to be public for this peer review?**  If you choose “no”, your identity will remain anonymous but your review may still be made public.

For information about this choice, including consent withdrawal, please see our Privacy Policy

Reviewer #1: No

Reviewer #2: No

**********

[NOTE: If reviewer comments were submitted as an attachment file, they will be attached to this email and accessible via the submission site. Please log into your account, locate the manuscript record, and check for the action link "View Attachments". If this link does not appear, there are no attachment files.]

**Figure Resubmissions:**

# Author comment: Author response to Decision Letter 1

**DOI:** 10.1371/journal.pgph.0007362.r003

16 Jul 2026

Attachment Submitted filename: Response to Reviewers.docx

# Peer review: Decision Letter 1

**DOI:** 10.1371/journal.pgph.0007362.r004

**Authors:** Julia Robinson

9 Sep 2026

Decolonising Global Health Evaluations: A Scoping Review of methodological frameworks

PGPH-D-26-01004R1

Dear Dr Hafez,

We are pleased to inform you that your manuscript 'Decolonising Global Health Evaluations: A Scoping Review of methodological frameworks' has been provisionally accepted for publication in PLOS Global Public Health.

Before your manuscript can be formally accepted you will need to complete some formatting changes, which you will receive in a follow up email. A member of our team will be in touch with a set of requests.

Please note that your manuscript will not be scheduled for publication until you have made the required changes, so a swift response is appreciated.

IMPORTANT: The editorial review process is now complete. PLOS will only permit corrections to spelling, formatting or significant scientific errors from this point onwards. Requests for major changes, or any which affect the scientific understanding of your work, will cause delays to the publication date of your manuscript.

If your institution or institutions have a press office, please notify them about your upcoming paper to help maximize its impact. If they'll be preparing press materials, please inform our press team as soon as possible -- no later than 48 hours after receiving the formal acceptance. Your manuscript will remain under strict press embargo until 2 pm Eastern Time on the date of publication. For more information, please contact globalpubhealth@plos.org.

Thank you again for supporting Open Access publishing; we are looking forward to publishing your work in PLOS Global Public Health.

Best regards,

Julia Robinson

Executive Editor

PLOS Global Public Health

***********************************************************

Reviewer Comments (if any, and for reference):

Reviewer's Responses to Questions

**Comments to the Author**

Reviewer #1: All comments have been addressed

Reviewer #2: All comments have been addressed

**********

publication criteria?

Reviewer #1: Yes

Reviewer #2: Yes

**********

3. Has the statistical analysis been performed appropriately and rigorously?

Reviewer #1: N/A

Reviewer #2: N/A

**********

4. Have the authors made all data underlying the findings in their manuscript fully available (please refer to the Data Availability Statement at the start of the manuscript PDF file)?

The PLOS Data policy

Reviewer #1: Yes

Reviewer #2: Yes

**********

5. Is the manuscript presented in an intelligible fashion and written in standard English?

Reviewer #1: Yes

Reviewer #2: Yes

**********

Reviewer #1: I'm looking forward to seeing this article published. Good work.

Reviewer #2: Thank you to the authors for their careful revision of the manuscript and for addressing the previous comments. The manuscript is generally well written; however, a final proofreading would help address any remaining minor grammatical or typographical errors. I have no further comments and believe the manuscript is suitable for publication.

**********

what does this mean?). If published, this will include your full peer review and any attached files.

**Do you want your identity to be public for this peer review?**  If you choose “no”, your identity will remain anonymous but your review may still be made public.

For information about this choice, including consent withdrawal, please see our Privacy Policy

Reviewer #1: No

Reviewer #2: No

**********
